# First GIS Analysis of Modern Stone Tools Used by Wild Chimpanzees (*Pan troglodytes verus*) in Bossou, Guinea, West Africa

**DOI:** 10.1371/journal.pone.0121613

**Published:** 2015-03-20

**Authors:** Alfonso Benito-Calvo, Susana Carvalho, Adrian Arroyo, Tetsuro Matsuzawa, Ignacio de la Torre

**Affiliations:** 1 Laboratory of Digital Mapping and 3D Analysis, CENIEH, Burgos, Spain; 2 Center for the Advanced Study of Human Paleobiology, The George Washington University, Washington, D.C., United States of America; 3 Interdisciplinary Center for Archaeology and Evolution of Human Behavior (ICArEHB), Universidade do Algarve, Faro, Portugal; 4 Institute of Archaeology, University College London, London, United Kingdom; 5 Section Language and Intelligence, Primate Research Institute, Kyoto University, Japan; CNR, ITALY

## Abstract

Stone tool use by wild chimpanzees of West Africa offers a unique opportunity to explore the evolutionary roots of technology during human evolution. However, detailed analyses of chimpanzee stone artifacts are still lacking, thus precluding a comparison with the earliest archaeological record. This paper presents the first systematic study of stone tools used by wild chimpanzees to crack open nuts in Bossou (Guinea-Conakry), and applies pioneering analytical techniques to such artifacts. Automatic morphometric GIS classification enabled to create maps of use wear over the stone tools (anvils, hammers, and hammers/ anvils), which were blind tested with GIS spatial analysis of damage patterns identified visually. Our analysis shows that chimpanzee stone tool use wear can be systematized and specific damage patterns discerned, allowing to discriminate between active and passive pounders in lithic assemblages. In summary, our results demonstrate the heuristic potential of combined suites of GIS techniques for the analysis of battered artifacts, and have enabled creating a referential framework of analysis in which wild chimpanzee battered tools can for the first time be directly compared to the early archaeological record.

## Introduction

The use of stone tools to crack open nuts by chimpanzees in West Africa has received considerable attention by primatologists [1–3], and the evolutionary implications of this behavior have been widely discussed [4–6]. Parallels between chimpanzee tool use and the archaeological record have been drawn [7–12], and in recent years the need for systematic comparisons between the two data sources has been widely recognized [13–16]. Modern humans that still use stone tools are also powerful analogs for understanding the evolution of technological behaviors [17]. There are, however, recent arguments stating the importance of also using *Pan troglodytes* as a model for understanding the origins of technology [18–20]. Early hominins and modern chimpanzees share some relevant features (e.g., brain size, arboreal adaptations, likely use of percussive technology) [21–23]. In addition, chimpanzees, regardless of variability in habitat type, group size, presence of predators, hunting behavior, etc, are all tool users [24]. More importantly, they have the largest and most complex repertoire of tool use apart from humans [25], with sequential use of tools [26] and use of tool-composites [11].

Stone tools used by wild chimpanzees have normally been associated with the presence of specific surface features such as depressions or concavities [4]. Pounding tools with similar features have been reported in archaeological assemblages such as Gesher Benot Ya´aqov (Israel), where presence of pitted stones has been associated with nut cracking activities [27]. Pitted stones have also been described in Olduvai Beds III and IV (Tanzania), although these have been linked to bipolar knapping activities [28] rather than to nut cracking. Similar tools have been also described in Upper Paleolithic and Mesolithic sites [29–31] as well in Holocene sites where, for example, Australian assemblages show that pitted stones can also be associated with shell fish processing [32].

An avenue for stone tool-use comparisons between chimpanzee and human ancestors is the application of archaeological perspectives to the West African chimpanzee stone tool assemblages, pioneered by Mercader et al [33–34]. More recent approaches have combined studies of such material culture with a direct observation of wild chimpanzee behavior [10–11], [35], in order to establish direct links between artifact patterns, site formation and chimpanzee technological behavior.

These pioneering works have paved the way for the use of an archaeological perspective to the study of chimpanzee stone tool use, in which nonetheless systematic analysis of battered artifacts is still lacking [36]. Recent progress on the study of battered stone tools in archaeological contexts [36–39] is leading to innovative approaches in this new field of research. However, despite the increasing amount of data available in Primatology and Archaeology, and the growing awareness of its relevance for understanding the evolution of technology in the human lineage (see [40–41] for a new round of analytical approaches to percussive technologies), the lack of systematic analysis of modern chimpanzee stone tools still precludes direct comparisons between the technological records of human and non-human primates.

The aim of this study is to present the first systematic analysis of modern nut-cracking tools (anvils/hammers) through the application of new GIS techniques to identify, grade and quantify damage patterns in the artifacts used by wild chimpanzees in Bossou (Guinea-Conakry). In addition, we present a variety of new analytical methods that will allow further quantitative cross-comparisons between human and non-human assemblages, and which will contribute to establishing a contextual framework in which the co-evolution of stone-tool technology in the human and chimpanzee lineages can be better understood.

## Materials

### Study site

The village of Bossou, in Guinea, West Africa, harbors a population of wild chimpanzees (*Pan troglodytes verus*) that have been systematically studied since 1976 [3], [42]. Bossou is one of eight long-term field sites for the study of chimpanzee behavior [15]. The core range of this community (comprised by 13 individuals during this data collection period) lies within the small forest of Bossou, and is between 5 and 7 km^2^. Bossou chimpanzees are especially known for making and using a large variety of tools [3], [25] and, more specifically, for the habitual use of a pair of movable stones (anvil and hammer stone) to crack open oil palm nuts (*Elaeis guinneensis*), which are abundantly available [3], [43–44]. There are only two West African sites where stone tool use by chimpanzees has been the focus of systematic studies: Bossou in Guinea and Taï in Ivory Coast. The chimpanzee community at Bossou represents a unique case study to examine the role of percussive technology in the evolution of technology: 1) this population does not customarily use boulders or wood as tools. The nut-cracking activities rely mostly on the use of movable stone tools [11]; 2) Stone tool dimensions are relatively ‘standardized’ [10], partially due to the species of nut that is cracked, i.e. the oil-palm, which is relatively soft [45]. Thus, tools are of small to medium size, and both the hammer and anvil along with the nuts are often transported to the nut-cracking sites [35].

### The outdoor laboratory and the experimental procedure

**Fig 1:**
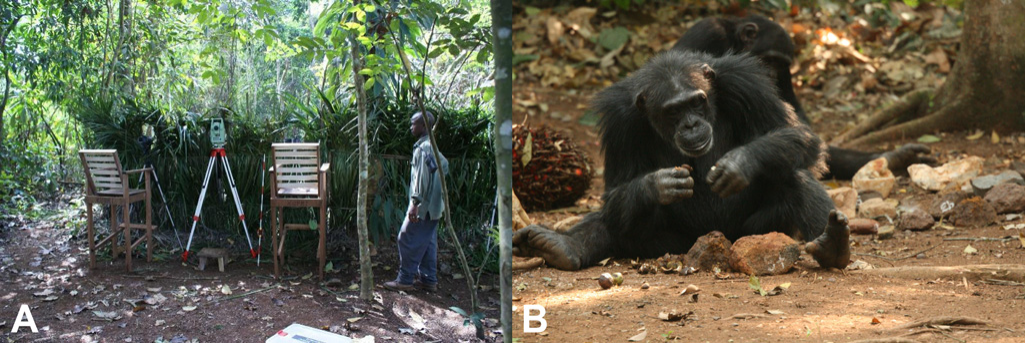
Bossou ‘outdoor laboratory’. A) View of the experimental area in the Forest of Bossou, Guinea. Researchers record the nut-cracking behavior every year, using several video cameras while staying behind a screen of vegetation, c. 20m distance from the wild chimpanzees using tools. B) Female using a stone hammer and anvil to crack open nuts. Note the assortment of stones on the right side which is provided by the researchers, along with the piles of nuts.

In 1988, a so-called ‘outdoor laboratory’ was initiated in the Bossou forest (Latitude 7°38'52.07"N, Longitude 8°30'17.95"W, WGS84), which is a part of the public National Research, with the permission of the Direction Nationale de la Recherche Scientifique et Technique (DNRS) and the Institute de Recherche Environnementale de Bossou (Republic of Guinea). This has been running for 26 years, providing exceptional insights concerning the development of tool use behavior and of the experimental assemblages used by these chimpanzees. All research involving wild chimpanzees was non-invasive and strictly adheres to ethics guidelines detailed by the Association for the Study of Animal Behaviour. This study (introduction of nuts and stones) is approved by the committee for the Ethical guideline of studying wild primates of the Primate Research Institute of Kyoto University (2013). Kyoto University has been directing all research conducted in this field site, in collaboration with the Guinea authorities since 1976. The ‘Outdoor Laboratory’ is a small cleared area on the top of a ‘sacred’ hill, within the core range of the wild chimpanzee community. Here, researchers place a matrix of numbered stones along with seven piles of nuts around the matrix, and wait behind a screen of vegetation for the chimpanzees to visit (Fig. 1 and see [10–11], [43], for more details of the outdoor methods). Each year, the nut cracking “experimental season” varies between December and February–this falls within the dry season and the peak of chimpanzee nut-cracking behavior [46]. The sample of stone tools analysed for this study was used by the wild chimpanzees during the nut-cracking season of December 2008 to February 2009, when 47 experimental sessions were conducted (totaling 34 h 48 min) using two species of nuts: *Elaeis guineensis* and *Coula edulis*, the latter being a non-locally available nut that has been introduced to this community a decade ago [35].

**Fig 2:**
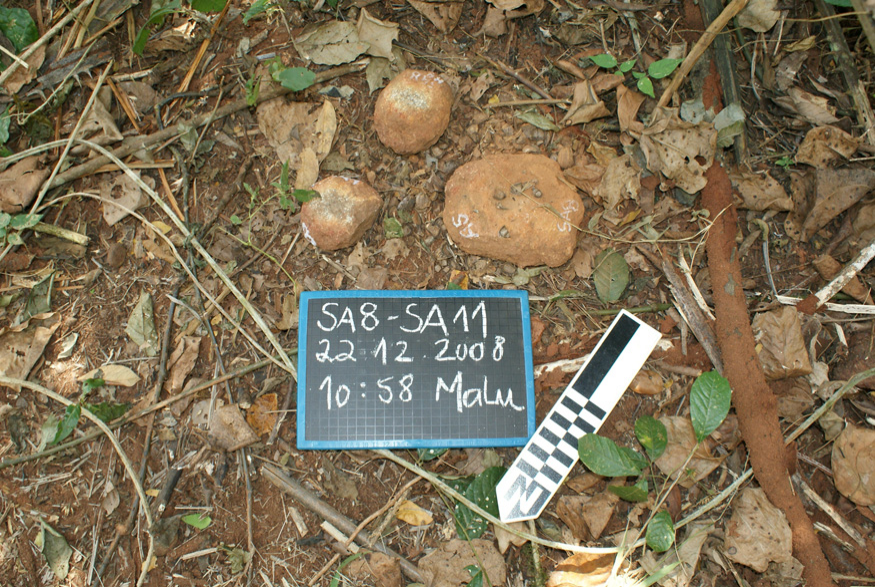
View of a natural nut-cracking site in Bossou forest, with tools 4 and 5 analysed in this study.

For the purpose of this study, the analysed sample of tools is a sub-set of anvils and/or hammer stones selected from the experimental assemblage (N = 4). Another small sample of stone tools (N = 2) used by the same chimpanzee population in a monitored natural nut-cracking site of the forest (Fig. 2) was analysed to compare with results from the experimental assemblage. Stone tools analysed were of local raw materials (amphibolite and African iron oxide).

Criteria to select stone tools for this study were: 1) artifacts that had indisputably been used for pounding activities by Bossou chimpanzees. 2) Objects representative of the raw materials usually available in the natural nut-cracking sites (e.g., African iron oxide and amphibolite). 3) Artifacts with clear battering marks susceptible of being analysed digitally and microscopically.

## Methods

Each battered surface was digitized, 3D scanned (3D laser scanner Next Engine) and analysed separately (8 battered surfaces out of 6 artifacts), following the GIS methods outlined in S1 Text. Identification of damage patterns in Bossou chimpanzee stone tools involved visual mapping [40] and automatized morphometric classifications. GIS morphometric classification [47–51] lacks initial input from the analyst, and grouping is based on an automatized GIS identification of variables, derived from 3D models scanned from the Bossou tools. In order to assess the validity of both techniques, visual mapping and morphometric classifications of the Bossou percussive tools were conducted independently, in such a way that the analyst of the morphometric classification had no access to the original artifacts but was provided with the 3D models suitable for the GIS study.

### Morphometric GIS analysis

Resulting Digital Elevation Models (DEM) of each scanned tool face (N = 8) were used for surface morphometric analysis of topographic attributes calculated with GIS (ArcGIS 10.1 and SAGA 2.1). Several Digital Surface Models (DSM) were calculated from elevation data (S1 Text), such as primary and secondary derivates (i.e. slope, aspect and curvatures), hillshading models, or topographic profiles [48–49]. These DSM were used for a first basic morphometric analysis of the stone tools surfaces. Subsequently, roughness [52–58], topographic position [50], [59], and relative depth DSM were calculated (S1 Text), in order to identify and interpret use-wear features. Roughness was used to estimate polish areas. We applied three different roughness indices: Terrain Ruggedness Index (TRI), Vector Ruggedness Measure (VRM), and the 3D/2D area ratio (see details in S1 Text). In addition, topographic position index (TPI) and relative depth models were applied to map and characterize surface depressions, in order to identify concavities associated with percussive activities.

### Visual mapping and GIS spatial pattern analysis

Following protocols outlined by de la Torre et al [40], digital images were geo-referenced in a local Cartesian system using ArcGIS 10.1. Macroscopically-identified percussive marks were outlined over the images, and indexes such as area, perimeter, and distribution and size of the areas covered by percussion marks (S1 Text), were calculated to produce a spatial pattern of the use wear distribution along the tools.

## Results

### Morphometric analysis

This analysis was based first on a basic morphometric study, in order to characterize the topography of stone tools surfaces. Then, we applied specific morphometric indices with a significant relevance in the identification of use wear features such us polish areas and depressions. Combining these indices, we generated a final morphometric classification which summarizes the spatial distribution of morphometric features for each artifact face.

#### Basic morphometric analysis

Results of basic morphometric analysis are shown in maps and tables of S1–S4. The statistical analysis of elevation of each battered face (S1 Fig. and S1 Table) indicates that artifacts 43 and 431 bear the highest mean elevations, followed by artifact 55, while artifact H4FB, H4, A/H55FB and A70 yield the lowest values. Slope distribution in stone tool faces indicates predominance of low-intermediate slopes (S2 Fig.), particularly in the 10.5º-31.1º range. There are few areas with slope values greater than 45º, which are mainly restricted to the artifact edges; only artifacts 43 and 431 (and to a lesser degree in artifact A3) consistently contain > 45º scarps across the surface. Artifacts A43 and A431 show the greatest mean slope, with values around 33–34º (S1 Table), while the rest of the objects have mean values between 23º and 26º. Curvature models (S3 Fig.) were simplified in a combined curvature model [60] (S4 Fig. and S2 Table). This model shows that predominant curvatures in the battered faces are V/V (convex profile and plan curvatures) and X/X (concaves plan and profile curvatures), with percentages respectively of 33.7% and 35.1%. Surfaces with X/V and V/X curvatures decrease to 15% of the cases, while the rest of the curvature cases are insignificant (≤ 0.1%) (S4 Fig.). Such proportions observed for all battered surfaces remain similar when percentages of curvature classes for each artifact are calculated (S4 Fig.).

#### Polish quantification: roughness indices

Polish in stone tool surfaces are usually associated with intensive use. Quantification of polish differences assists to recognize areas of the stone surface more heavily used, and to discriminate used from unused areas. Polish was estimated through different roughness indices: TRI, VRM and 3D/2D area ratio indices (S1 Text). The three resulting roughness models for the artifacts are shown in S5 and S6 Figs., where values has been calculated for each cell considering the closest neighborhood (radius = 0.1 mm). Results are consistent and indicate a broadly similar roughness distribution, with areas of low roughness and high roughness on similar positions across the three models. There are nonetheless some differences (S1 Table): while artifacts 43 and 431 (African iron oxide) display the highest mean values in the three roughness models, the rest of tools show variable patterns in the TRI model and (particularly) the VRM model, which capture small roughness differences among the battered surfaces (S5 Fig. and S1 Table). The TRI model suggests that artifacts A/H55FB and H4 share nearly the same lowest mean values, but the VRM model positions H4 as the tool with the lowest roughness. A3, A70 and H4FB pieces present intermediate values.

**Fig 3:**
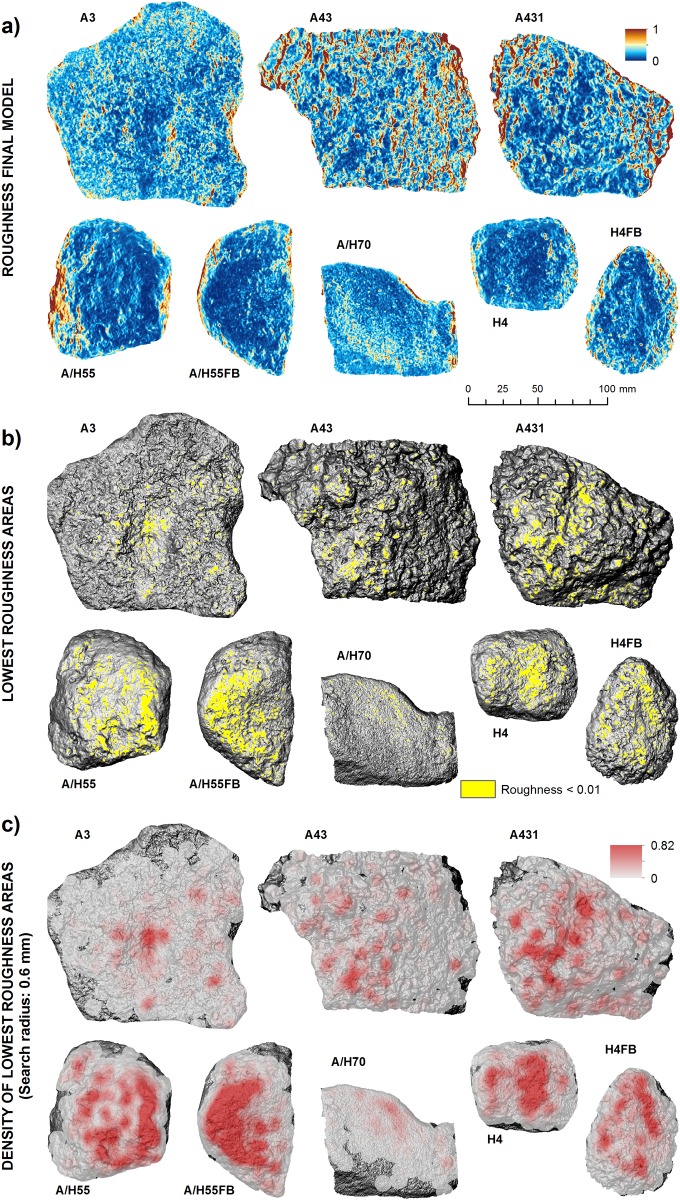
Roughness model applied to the identification of polished areas. A). Roughness final model derived from the combination of roughness models TRI, VRM and 3D/2D area ratio. Lowest roughness areas (values<0.01) identified in the roughness final model. Density map of lowest roughness areas, computed using a search radius of 0.6 mm.

The combined or final roughness model showed in Fig. 3a synthesizes variability of the three roughness models (TRI, VRM and 3D/2D area ratio). From this model, we have derived the lowest roughness areas (value<0.01), which represent the distribution of the most polished areas in each piece (Fig. 3b). Polished areas are more abundant in tools A/H55FB, H4FB and then A431, while the lowest percentage of polished areas is in A70 (Table 1). To enhance visibility of the distribution of polished areas, a density map was calculated using a search radius of 0.6 mm (Fig. 3c), which shows in red the areas where density of polished spots is highest.

**Table 1:** Percentage of lowest roughness areas in stone tool surfaces.

Values of Final Roughness Model <0.01			
*Anvil*	*Count*	*Area (mm2)*	*%*
A3	40984	409.84	8.90
A43	47518	475.18	10.32
A431	81947	819.47	17.79
A/H55	91582	915.82	19.89
A/H55FB	94128	941.28	20.44
A/H70	10806	108.06	2.35
H4	50705	507.05	11.01
H4FB	42886	428.86	9.31

#### Depression identification: TPI and relative depth models

**Fig 4:**
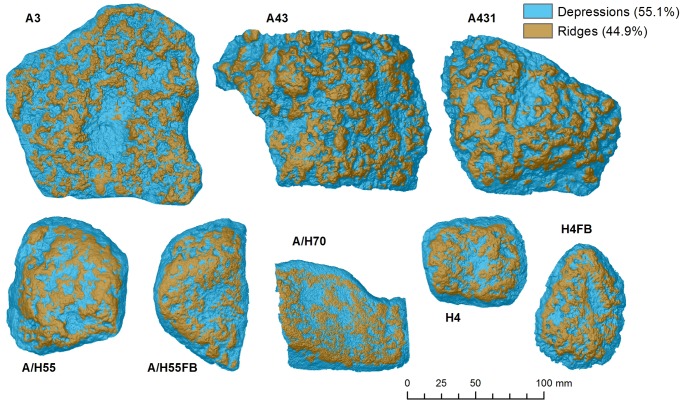
Distribution of depressions and ridges in artifact surfaces, calculated from the classification of models TPI 0.5 mm and TPI 1 mm (S6 Fig.).

Depressions are key features produced during nut cracking activities. In this work, automatic identification of depressions and their characterization were calculated using TPI, relative depth models (S1 Text) and topographical profiles. Since TPI is a scale-dependent phenomenon, it can be used to identify depressions of different size, according to the scale of measurement. In S7 Fig., the TPI index was calculated considering a neighborhood of 0.1, 0.5 and 1 mm. TPI 0.1 mm and TPI 0.5 mm maps show smaller depressions and ridges of the tool surface, whereas TPI 1 mm shows more regional depressions. Lowest values of TPI 0.5 mm and TPI 1 mm were then combined to classify the artifact ridges (where TPI>0) and depressions (where TPI<0). The resulting map includes both small and large depressions and ridges across the battered surface (Fig. 4).

TPI analysis indicates that in general, depressions are slightly more abundant than ridges, covering 55.1% of the surface of tools (Fig. 4, Table 2). This ratio is similar in every artifact (Table 2) except for objects A3 and A431, which are dominated by large depressions (Fig. 4), and where the proportion of depressions to ridges is slightly higher (57.8% and 56.7% respectively, Table 2). Depressions are usually characterized by higher slopes (mean slopes of 31º) and higher roughness (VRM = 0.10, TRI = 0.06 and 3D/2D area ratio = 1.36) (S3 Table), associated with scarps and slopes contained within the depressions. Ridges display mean slopes of 26º, and roughness values of VRM = 0.07, TRI = 0.04 and area ratio = 1.21). S4 Table shows the statistical distribution of elevation, slope and roughness in the depressions and ridges for each artifact.

**Table 2:** Percentage of depressions and ridges in stone tool surfaces.

*Anvil*	*Zone*	*Area (mm2)*	*%*
A3	Depression	9561.40	57.58
	Ridge	7042.82	42.42
A43	Depression	7822.92	54.78
	Ridge	6458.69	45.22
A431	Depression	6584.98	56.57
	Ridge	5055.55	43.43
A/H55	Depression	3765.28	52.17
	Ridge	3452.10	47.83
A/H55FB	Depression	3132.71	52.55
	Ridge	2828.57	47.45
A/H70	Depression	3441.43	54.61
	Ridge	2860.39	45.39
H4	Depression	2365.97	53.46
	Ridge	2059.97	46.54
H4FB	Depression	2477.99	53.19
	Ridge	2181.15	46.81

**Fig 5:**
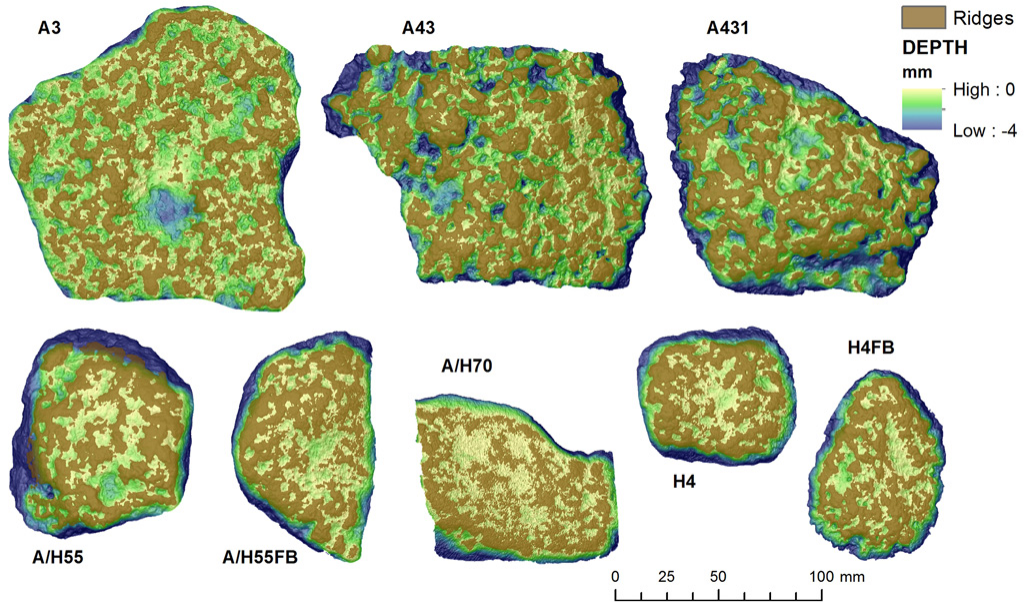
Relative depth model of artifact surfaces. These models calculated by subtracting the DEM from TPS models (Top Potential Surface), which represents the interpolated surface joining the highest points of the ridges.

Relative depth models (Fig. 5) indicate different patterns in the objects. Artifacts A3, A43 and A431 show the deepest depressions (depth<-4mm), while pieces A/H55, A/H55FB, H4 and H4FB feature intermediate values around-2 mm. Artifact 70 yielded the lowest values, with depressions averaging -1mm.

#### Morphometric classification

**Fig 6:**
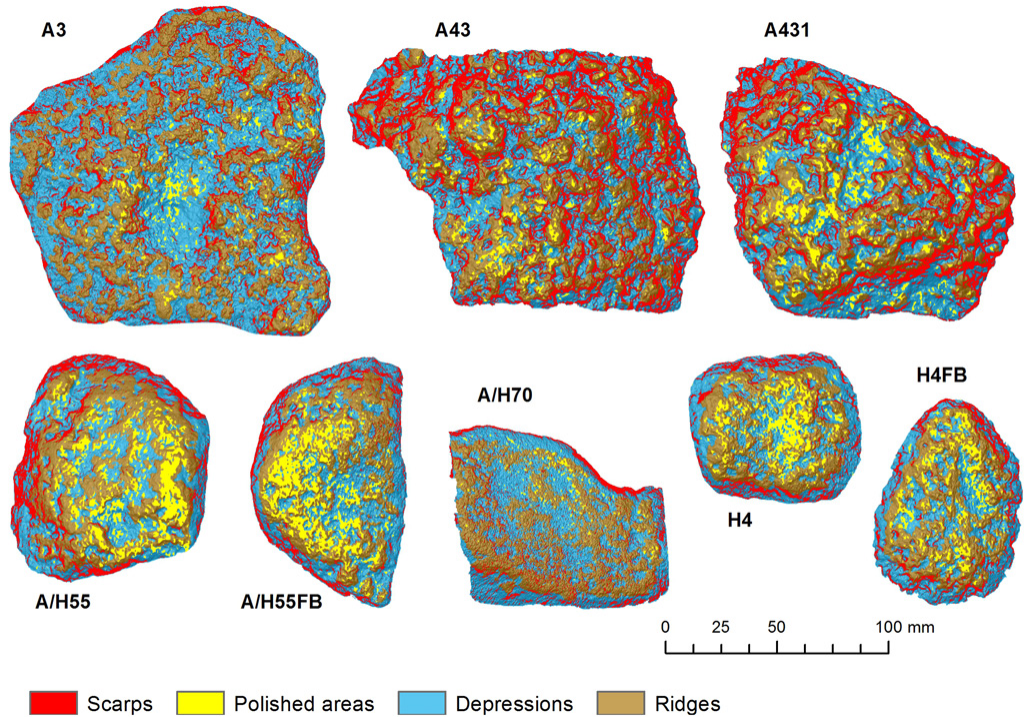
Morphometric classification of the artifacts, based on slope, roughness and TPI results. Identified morphometric features in this classification include ridges and depressions, polished areas (values of the roughness final model <0.01), and scarps (slope > 45º).

A morphometric classification of the artifacts based on slope, roughness and TPI results is shown in Fig. 6. This classification provides the spatial patterns and distribution of surface morphometric features for each artifact, including ridges and depressions, polished areas (lowest roughness areas, roughness <0.01), and scarps (slope > 45º). When polished areas and depression/ridges are superimposed, it is observed that polished areas develop in ridges as well as in depressions, but are much more abundant in ridges (Table 3). For example, 75.8% of the polished areas in artifact A43 are located in ridges, with a similar trend (70.1%) in artifact A/H55FB. This pattern only changes in artifact A3, where polished surfaces are less abundant in ridges, due to a large depression in the center of the anvil that contains most of the polished areas (Fig. 6).

**Table 3:** Percentage of lowest roughness areas (values of roughness final model <0.01) in the depressions and ridges identified in the artifact surfaces using the Topographical Position Index (TPI).

*Anvil*	*Depression*	*Ridge*
	*%*	*%*
A3	54.3	45.7
A43	24.2	75.8
A431	42.6	57.4
A/H55	35.7	64.3
A/H55FB	29.9	70.1
A/H70	44.2	55.8
H4	38.8	61.2
H4FB	34.0	66.0

### Visual identification and GIS spatial pattern analysis

**Fig 7:**
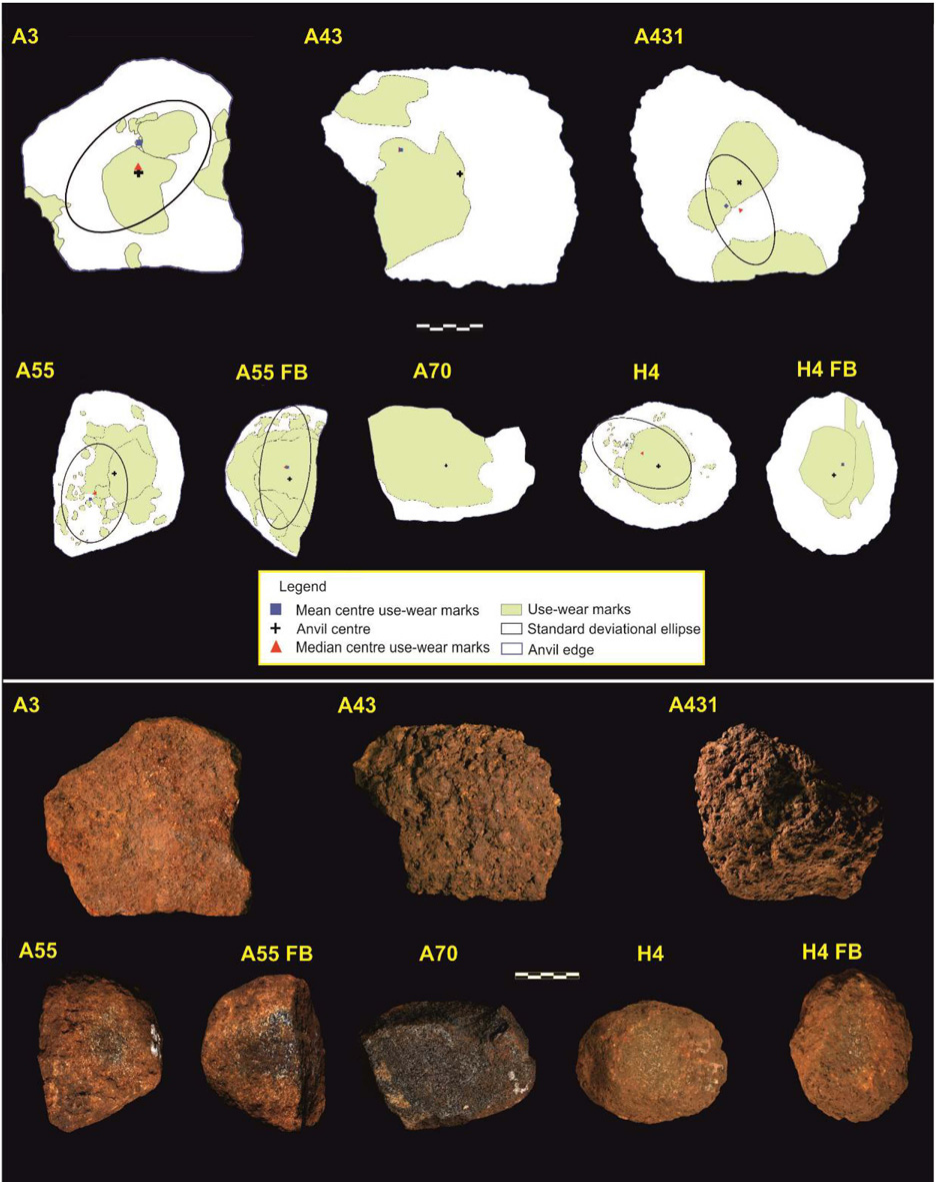
Visual mapping of battering damage in stone tool surfaces.

Distribution of use wears produced by visual mapping is shown in Fig. 7, and their spatial analysis in Table 4. Visual analysis of the Bossou stone tools revealed the presence of depressions located on one horizontal plane on one tool from SA8 Site (A3), and two from the Outdoor Lab (43 and 431). Depressions were considered here as concavities developed on the stone surface as a result of a fatigue wear mechanism producing a plastic deformation due to a thrusting percussion movement. All these tools show development of a double depression characterized by a concave cross section, suggesting the presence of two working areas where the nut was repeatedly placed. These depressions show a centered location except on tool #43, where they are closer to the edge. In addition, it is relevant to note that all depressions are located on a single horizontal plane of the blanks, indicating absence of tool rotation.

**Table 4:** Indices used to characterize the spatial pattern distribution of use wears identified visually, following GIS protocols outlined by de la Torre et al [25].

Index	Unit	A3	H4	H4 FB	A43	A431	A55	A55 FB	A70
**Tool**									
Area	cm^2^	200.29	89.57	91.60	250.20	205.87	95.74	71.51	82.39
Perimeter	cm	56.3	35.4	36.2	65.5	57.2	37.1	33.9	37.0
**Use Wear**									
Quantity	nº	11	32	2	2	3	31	11	1
Area	cm^2^								
Maximum		25.09	23.67	18.43	53.02	22.84	12.24	23.28	55.47
Mean		4.83	8.11	14.98	35.275	17.45	1.20	5.05	55.47
Minimum		0.30	0.01	11.52	17.53	7.15	0.02	0.22	55.47
Total		53.12	26.77	29.95	70.55	52.34	37.31	55.53	55.47
Perimeter	cm								
Maximum		20.0	17.9	25.9	19.4	21.8	21.9	24.2	30.1
Mean		7.9	1.7	21.0	26.2	16.6	34.5	9.9	30.1
Minimum		2.5	0.4	1.6	19.4	10.1	0.6	1.9	30.1
Total		86.7	54.3	42.0	52.4	49.8	107.1	109.1	30.1
PA	%	26.52	29.89	32.70	28.20	25.42	38.97	77.65	67.33
LUW	%	12.53	26.43	20.12	21.19	11.09	12.78	32.55	67.33
D	cm^2^	0.05	0.36	0.02	0.01	0.01	0.32	0.15	0.01
ED	cm^-1^	0.43	0.61	0.46	0.21	0.24	1.12	1.53	0.37
MNSH	ad.	1.33	1.22	1.61	1.29	1.14	1.21	1.48	1.14
DAC	cm								
Minimum		0.00	0.00	0.00	0.00	0.00	0.00	0.00	0.00
Mean		3.76	3.37	0.74	2.65	1.88	3.16	3.02	0.00
Maximum		7.18	5.46	1.47	5.30	4.16	5.70	5.04	0.00
DAE	cm								
Minimum		0.00	0.00	0.10	0.00	0.00	0.20	0.00	0.00
Mean		2.35	1.95	2.20	2.55	2.76	1.96	1.05	0.62
Maximum		5.89	3.26	3.73	7.01	6.18	4.22	3.32	2.93
**Standard deviational ellipse**									
XstdD	cm	3.60	2.33	n/a	n/a	2.19	3.72	5.13	n/a
YstdD	cm	6.30	4.00	n/a	n/a	4.31	2.44	2.05	n/a
Area	cm^2^	71.31	29.25	n/a	n/a	29.59	28.46	33.11	n/a
Elongation	ad.	1.75	1.72	n/a	n/a	1.97	1.52	2.50	n/a
EMNC-MDC	cm	1.75	1.29	0.00	0.00	1.06	0.53	0.19	n/a
EMNC-AC	cm	0.46	1.60	1.06	4.84	2.02	2.10	1.00	n/a
AMNC-AE	cm	6.30	3.29	3.93	3.65	5.00	3.02	2.58	n/a

A second group of tools is formed by two tools from the Outdoor Lab (A/H55, and A70) and one from the SA 8 Site (H4). At Bossou, rocks usually bear an oxide coating developed during soil burial and on these three tools. This oxide coating is located on horizontal planes of the blanks, the one that will be modified as a result of the use of the blanks. The use-wear formation process on these tools begins when, on a first stage, the oxide coating is rapidly eroded out due to its friable properties, process that is abruptly interrupted when the core of the stone is exposed. If the activity continues, a second stage of use wear formation begins, producing a modification of the crystals that form the rock matrix, with presence of microfractures along the crystals. Although a large scale modification of the surface has not been identified microscopically, where the inner part of the blank is exposed there are some areas with crystal crushing produced by the surface contact between the active and the passive element. Even though this coating erosion areas show similar morphology to a depression, they were considered here as a distinct group, as use wear is limited to the coating layer and does not modify the rock surface. In addition to these features, most of these tools bear impacts marks on their surfaces.

Main characteristics of the main damage patterns are shown in Table 5. Regarding the size of use wear areas, the largest PA indexes are observed in tools A/H55 and A/H70, followed by A/H55FB and A70, while the rest of the tools have <40% PA values. This pattern is similar in the largest use wear marks (LUW), where tools A70 and A/H55FB again show the highest values (LUW> 30%) (Table 4).

**Table 5:** Main characteristics of damage patterns, compared with the morphometric depressions, ridges and lowest roughness areas (polished areas).

A/H55FB	Passive/Active	Oxide coating erosion*	battering around the eroded area	32.55	Centred	52.55	47.45	29.9	70.1
A/H70	Passive/Active	Oxide coating erosion*		67.33	Centred	54.61	45.39	44.2	55.8
H4	Active	Oxide coating erosion*	battering around the eroded area	26.43	Centred	53.46	46.54	38.8	61.2
H4FB	Active	Oxide coating erosion*	battering around the eroded area	20.12	Centred	53.16	46.81	34.0	66.0

* PA-data only from depressions

* Pa data only with totally eroded areas

The highest values of the D index correspond to H4 and A/H55, consistently with the ED index, where battered surfaces A/H55FB (ED = 1.53 cm^-1^) and A/H55 (ED = 1.12 cm^-1^) are ranked first. An ED value < 0.65 cm^-1^ in the rest of battered surfaces indicates an overall low density of use wear. This low density is related with high elongation values; battered surfaces A3, H4 and A/H55 present similar elongation values (between 1.75 and 1.39), with the highest elongation values in A431 (elongation = 1.97) and A/H55FB (elongation = 2.50).

With concern to MNSH, use wear marks show similar indexes (MNSH<1.30) except in three battered surfaces (A3, H4FB and A/H55 FB), with values over 1.30 suggesting the presence of marks with an elongated morphology. Mean DAC values are relatively high (DAC<4.0 cm), although all tools possess clusters of marks with a centered location. In fact, when looking at the AMNC-AC index, all tools show low AMNC-AC values (<2.50 cm) except A43 (AMNC-AC = 4.84 cm).

The DAE index yields homogeneous results, with the highest value in the battering surface A431 (DAE = 2.76cm), and the minimum in A/H70 (DAE = 0.62 cm). A similar pattern exists in the AMNC-AE index, where A3 (AMNC-AE = 6.30 cm) and A431 (AMNC-AE = 5.00 cm) show the highest values. The DAE index values (see details for each tool in Table 4) show marks located off center and close to the blank edges, which highlights the distribution of damage not only in the center but also around the edge of the battering surfaces (Fig. 7).

## Discussion

**Fig 8:**
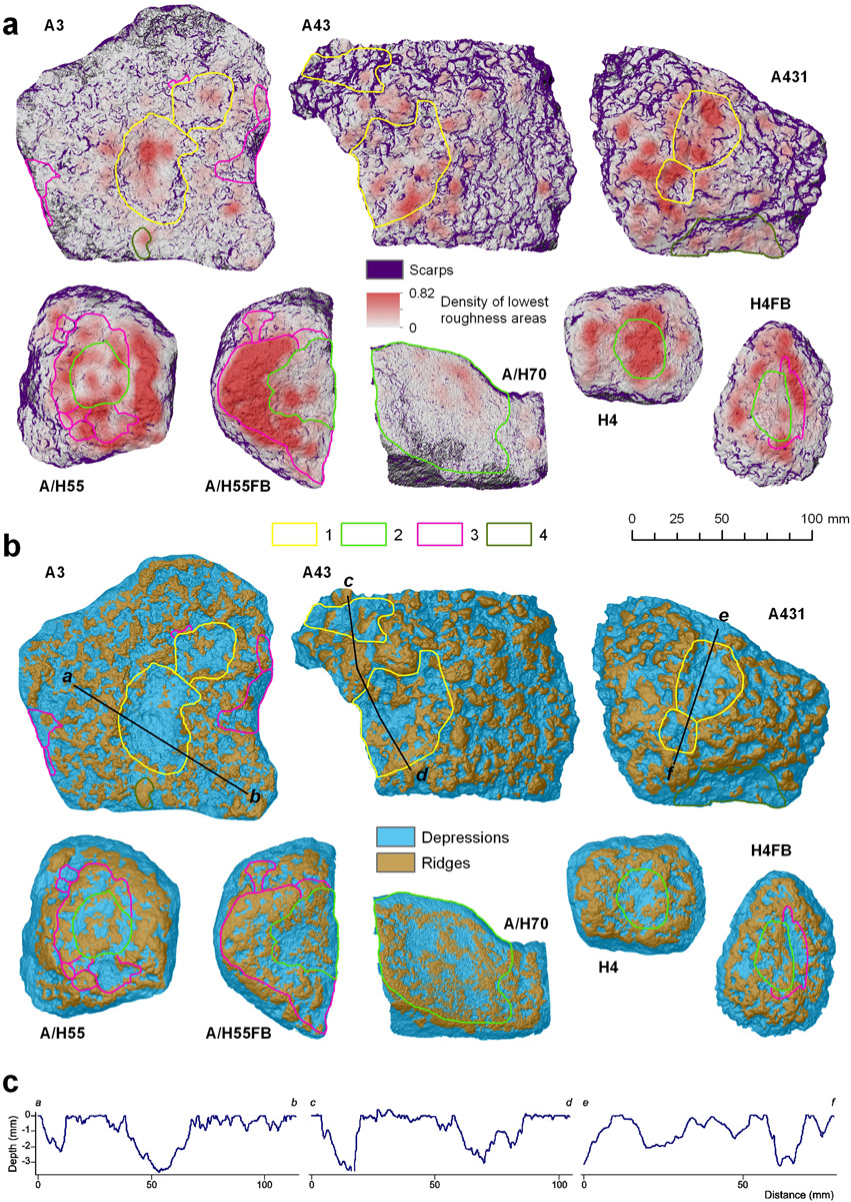
Comparison of wear damage in visually and morphometrically-derived features from Digital Surface Models. A) Comparison of main use wears with density of lowest roughness area. B) Comparison of the morphometric classification of depressions and ridges with depressions identified visually. C) Topographic profiles using the relative depth models from depressions identified in pieces A3, A43 and A431. Main use wears identified visually: 1, Depressions; 2, Oxide coating erosion; 3, Impact or battering marks; 4, Polish areas.

General topographic characterization of battered surfaces using slope, roughness and elevation (S1 Fig.) group the pieces in two sets. The first group includes artifacts A43 and A431, which show the greatest topographic variability, characterized by the highest mean values of roughness and slopes (S1 and S4 Tables, S5 and S6 Figs). Morphometric similarities between A43 and A431 are consistent, for both pieces derive from the same block of African iron oxide, which fractured during nut cracking. On the other hand, slope and roughness variables display lower-and often similar- mean values in the rest of artifacts (S1 Table): these variables make up a second group characterized by flatter and smoother faces, typical of amphibolites with an oxide coating. Fig. 8 compares visual and morphometric classifications of the battered surfaces, and demonstrates the consistency of both methods in grouping artifacts in two sets. This visual correspondence is also backed statistically, as shown in Tables 5, 6 and 7.

**Table 6:** Percentages of statistical occurrence of the morphometric classes in visually-identified use wear.

		MORPHOMETRIC CLASSES							
Use wear feature	Tool ID	Roughness<0.01*	Roughness<0.01**	Scarps (>45º) *	Scarps (>45º) **	Depressions*	Depressions**	Ridges*	Ridges**
		%	%	%	%	%	%	%	%
Depressions		***36*.*0***	***8*.*0***	***12*.*7***	***14*.*1***	**21.6**	**67.5**	**13.4**	**32.5**
	A3	43.6	6.4	12.5	8.8	22.6	77.5	8.9	22.5
	A43	21.3	5.4	17.5	20.9	25.4	61.8	19.0	38.2
	A431	54.8	15.8	6.0	10.1	15.4	61.7	12.5	38.3
Oxide coating		***22*.*4***	***8*.*4***	***13*.*2***	***5*.*4***	***27*.*0***	***52*.*8***	***27*.*4***	***47*.*2***
erosion	A/H55	15.3	15.6	0.8	0.9	11.9	50.1	13.0	49.9
	A/H55FB	11.5	8.3	10.6	4.6	25.9	61.7	17.8	38.3
	A/H70	86.1	2.2	48.8	8.1	63.0	51.7	70.6	48.3
	H4	49.5	33.6	0.9	0.5	18.2	57.5	15.4	42.5
	H4FB	13.5	9.3	2.5	2.0	10.0	39.7	17.3	60.3
Batterings marks		***46*.*1***	***21*.*4***	***7*.*6***	***5*.*5***	***14*.*1***	***47*.*4***	***19*.*3***	***52*.*6***
	A3	3.7	1.8	17.1	0.0	6.1	68.8	3.7	31.2
	A/H55	30.8	18.3	10.4	10.7	20.4	50.0	22.3	50.0
	A/H55FB	79.5	29.9	7.6	2.4	30.8	38.6	54.5	61.4
	H4FB	20.8	23.5	0.2	10.3	9.7	63.4	6.4	36.6
Polish		***8*.*9***	***7*.*1***	***4*.*1***	***17*.*1***	***5*.*6***	***80*.*2***	***1*.*8***	***19*.*8***
	A3	2.9	9.6	0.2	3.0	0.2	16.1	1.5	83.9
	A431	14.1	6.7	0.0	0.0	13.3	88.3	2.3	11.7

* Percentage calculate with respect to total morphometric class area in the anvil

** Percentage calculate with respect to the use wear area

**Table 7:** Topographic characterization of visually-identified use wear, using morphometric variables extracted from the DSM.

		MORPHOMETRIC VARIABLES				
Use wear feature	Tool ID	Slope	Curv. Profile	Curv. Plan	Roughness	Depth
		º	1/mm	1/mm	d.u.	mm
		Mean	Mean	Mean	Mean	Mean
Depressions		***27*.*6***	***-1*.*29***	***-3*.*39***	***0*.*080***	***-1*.*09***
	A3	24.9	3.42	0.35	0.069	-0.97
	A43	31.2	-5.59	-7.11	0.096	-1.40
	A431	25.3	-0.88	-2.45	0.067	-0.69
Oxide coating erosion		***21*.*7***	***-4*.*53***	***-1*.*74***	***0*.*061***	***-0*.*21***
	A/H55	19.6	-1.10	-0.03	0.040	-0.19
	A/H55FB	21.7	-5.31	-4.19	0.066	-0.36
	A/H70	23.7	-5.58	-1.17	0.071	-0.20
	H4	14.7	-0.76	-1.62	0.026	-0.15
	H4FB	19.7	-5.30	-2.94	0.052	-0.12
Batterings marks		***20*.*8***	***-3*.*02***	***-0*.*54***	***0*.*050***	***-0*.*40***
	A3	31.4	-5.46	-0.15	0.112	-1.07
	A/H55	21.8	-1.53	0.12	0.046	-0.37
	A/H55FB	17.2	-4.01	-0.42	0.034	-0.23
	H4FB	16.9	2.86	-0.06	0.037	-0.17
Polish		***30*.*4***	***-1*.*30***	***-0*.*19***	***0*.*048***	***-2*.*16***
	A3	17.5	-6.39	3.32	0.039	-0.12
	A431	***32*.*1***	-0.66	-1.03	0.049	-2.42

In Fig. 8, combination of polish areas and depressions also allows to identify additional patterns. Distribution patterns of lowest roughness areas are closely linked to use-wear features identified visually, and to their functionality. Thus, active and mixed (active/passive) pieces show a higher percentage of lowest roughness areas in ridges (61–70%, Table 5), with concentric or semi-concentric distributions around internal areas (Fig. 8a). This pattern is clear in pieces A/H55, A/H55FB, H4FB and H4. These internal areas usually coincide with oxide coating erosion areas, such as in pieces A/H55, A/H55FB and H4FB (Fig. 8a). Oxide coating erosions are characterized by intermediate values of slope and roughness, and show the shallowest depressions (Table 7). Within the active and mixed action pieces, only artifact A/H70 differs substantially, as it is characterized by high roughness values (VRM = 0.07, Table 7) and a very low frequency of lowest roughness areas. In contrast to the active/mixed pattern, passive pieces A3, A43 and A431 show a more disperse distribution of lowest roughness areas (Fig. 8a). Lowest roughness areas were 44–55% concentrated within the depressions identified visually, which were generated by plastic deformation during battering processes. In addition, visually-identified depressions match with morphometric depressions calculated using the TPI index (61–77%, Table 6, Fig. 8b). In such depressed areas, scarps are abundant (Table 6) and show the greatest depths (Table 7), from -2 mm to -5 mm (Fig. 8c).

Concentric and semi-concentric lowest roughness areas also match with the impacts or battering belts identified visually in the active and active/passive pieces A/H55, A/H55FB, H4FB (Fig. 8b), in some cases with statistical significance of 79.5% (piece A/H55FB, Table 6). Impact areas also are associated with morphometric depressions, which include partial lowest roughness areas. These impact depressions are recorded in the TPI index classification (Fig. 8b), where piece H4FB bore circular morphologies (MNSH = 1.1) and significant mean depths of-2 mm, whereas artifacts A/H55FB and H4FB show shallower elongated or irregular impact depressions (depth <-0.9 mm, MNSH = 1.5). In general, impacts in active pieces produce the lowest mean slopes (20.8 mm, Table 7), containing low percentage of the artifact scarps (7.6%, Table 6).

Observed impacts areas in the passive artifacts are scarce, and are identified only in piece A3 (Figs. 8a and b). These impacts areas are distributed mainly around the anvil edge, and respond to a low percentage of lowest roughness areas (<4%, Table 6). These impact areas are often associated with elongated depressions, probably produced by fragments detached from the anvil (Fig. 8b).

Polish use wear mapped during the visual classification is limited to anvils A3 and A431. Although these areas show low roughness (mean VRM = 0.048, Table 7), conversely they exhibit the lowest percentages of lowest roughness areas (around 8.9%), high slopes (30.4º), and only slightly concave surfaces (Table 7). Such values are biased by the polish wear mapped in A431, which is located in the edge of the anvil.

In summary, our results indicate that morphometric patterns in active pieces are defined by higher percentages of lowest roughness areas located in ridges, which form semiconcentric flat belts that co-occur with impact marks and delimit erosion internal areas. Passive pieces tend to show a more scattered distribution of lowest roughness areas, which are locally concentrated in big depressions and characterized by greater depths and steeped edges. In addition, polish preferentially develops in soft raw materials such as iron oxide or over the friable coating of other rocks, and is found both in active and passive pounders.

Further interpretation of the use wear patterns requires a step beyond the results of the morphometric GIS analysis, and dwell into the behavioral data collected during the experiments. Although such analysis is still ongoing, it can be speculated here that the abundance of marks around the edge of battered artefacts (see DAE index values) rather than around the center (as expected from the location of the nut in the middle part of the anvils), is explained either by juvenile chimpanzees still not mastering nut-cracking, and/ or due to the repetitive contact between the hammer and the edge of the anvil when chimpanzees rest hammers in the intervals between nut-cracking motions. In addition, it is likely that the deep depressions reported in some of the anvils correspond to their prolonged use life by some individual chimpanzees. These and other aspects (for example, the possibility that experienced adults leave a distinct signature on the tools) should be considered in future analysis, in which the morphometric proxies developed in this paper shall serve as the basis to interpret behavioral data retrieved during etho-archaeological studies.

The present study has contributed to discriminate signatures of passive and active pounders, and to develop reliable quantitative parameters of battering damage identification that have been validated through blind tests of morphometric and visual analysis, and corroborated with the behavioral data. As such, our results contribute to set the foundations for a quantitative approach to the study of the Bossou pounding tools, which should next be combined with other perspectives in order to provide a comprehensive view of wild chimpanzee stone tool technology. Long-term field studies of wild chimpanzees in the Bossou [3] and Tai forests [1] are providing an invaluable source of data to understand stone technology among extant primates. Increasing awareness of the archaeological potential of the primate material culture to inform human evolution has led to pioneering studies of chimpanzee stone tool flaking [61], site excavation [33–34] and chaînes opératoires [10].

## Conclusions

Our results demonstrate the potential of morphometric analysis, especially using variables such as roughness methods (especially VRM index), TPI index, and relative depth models. These were calculated through automatic morphometric classification of 3D models in order to identify use wear patterns in stone tools, and validated through the visual mapping of battered areas. These variables can be calculated automatically, providing an accurate and objective way to analyze morphological features and their spatial distribution in archaeological, experimental and primatological stone tools. Furthermore, these methods are applicable not only to meso-scale 3D scans, but also to microtopographic models, and can be used to establish patterns of tool damage and use wear in lithic assemblages.

The newly emerged field of Primate Archaeology aims establishing analytical foundations to interpret primate behavior from an archaeological viewpoint [6], [45], [62–64], but much work is still needed to develop appropriate comparative protocols with the archaeological record. This paper has presented the first systematic GIS analysis of stone tools used by modern wild chimpanzees during nut-cracking activities, and in doing so has also provided innovative analytical techniques that can be applied to the early human archeological record, therefore prompting comparisons. Further ongoing research is focused on the comparison between the microscopic and technological analysis of the assemblage and the behavioral data collected chimpanzee nut-cracking (Carvalho et al. in prep). The morphometric classification of battering use wear, validated through blind testing by visual inspection, provides an enhanced method for the study of material culture and will strengthen the links between the behavior of extant primates and the archaeological record of our early human ancestors.

## Supporting Information

S1 Fig Digital Surface Models (DSM). Digital Surface Models showing the elevation distribution in the stone tools faces. a) Continuous distribution of the elevation. b) Elevation distribution classified in ten groups defined by the statistical natural breaks of the data according to the Jenk´s method (ArcGIS 10.2.1).
(JPG)

S2 Fig Digital Slope Models. Digital Surface Models showing the slope distribution in the stone tools faces. Slope measures the rate of change of elevation in the direction of steepest descent. a) Continuous distribution of the slope. b) Slope distribution classified in ten groups defined by the statistical natural breaks of the data according to the Jenk´s method (ArcGIS 10.2.1).
(JPG)

S3 Fig Curvature models. Digital Surface Models showing the curvature distribution in the stone tools faces. Curvature is the second derivate of the elevation, calculated following the steepest direction (profile curvature), its perpendicular (plan curvature), or a sum of both (tangential or total curvature).
(JPG)

S4 Fig Curvature classification. Curvature classification of the stone tools topography based on Dikau 1989, using profile and plan curvatures.
(JPG)

S5 Fig Digital roughness models (neighborhood = 0.1 mm). Digital Surface Models showing the roughness distribution in the stone tools faces, estimated through the Terrain Ruggedness Index (TRI), the Vector Ruggedness Measure (VRM), and the 3D/2D area ratio. Roughness models calculated considering the minimum neighborhood (radius = 0.1 mm).
(JPG)

S6 Fig Digital roughness models (neighborhood = 0.5 mm). Digital Surface Models showing the roughness distribution in the stone tools faces, estimated through the Terrain Ruggedness Index (TRI), the Vector Ruggedness Measure (VRM), and the 3D/2D area ratio. Roughness models calculated considering a wider local neighborhood (radius = 0.5 mm).
(JPG)

S7 Fig Topographic Position Index models. Digital Surface Models showing the value distribution of the Topographic Position Index in the stone tools faces, estimated considering neighborhood radius of 0.1 mm (a), 0.5 mm (b) and 1 mm (c).
(JPG)

S1 Table DSM basic statistics of stone tools. Elevation (mm), slope (degrees) and roughness (dimensionless) basic statistics of the stone tools, estimated from DSM.
(DOC)

S2 Table Curvature characteristics of depressions and ridges. Percentage of curvature classes in the depressions and ridges for every stone tool face.
(DOC)

S3 Table DSM basic statistics in the depression and ridges of the stone tools. (DOC)

S4 Table DSM basic statistics in the depression and ridges for every stone tool. (DOC)

S1 Text GIS Methodology. Morphometric analysis and spatial pattern analysis from visual identification.
(DOCX)
