# Supplementary figures and images for: First GIS Analysis of Modern Stone Tools Used by Wild Chimpanzees (Pan troglodytes verus) in Bossou, Guinea, West Africa
Source: PLoS One. 2015 Mar 20;10(3):e0121613. doi: 10.1371/journal.pone.0121613 (PMC4368754; doi:10.1371/journal.pone.0121613)

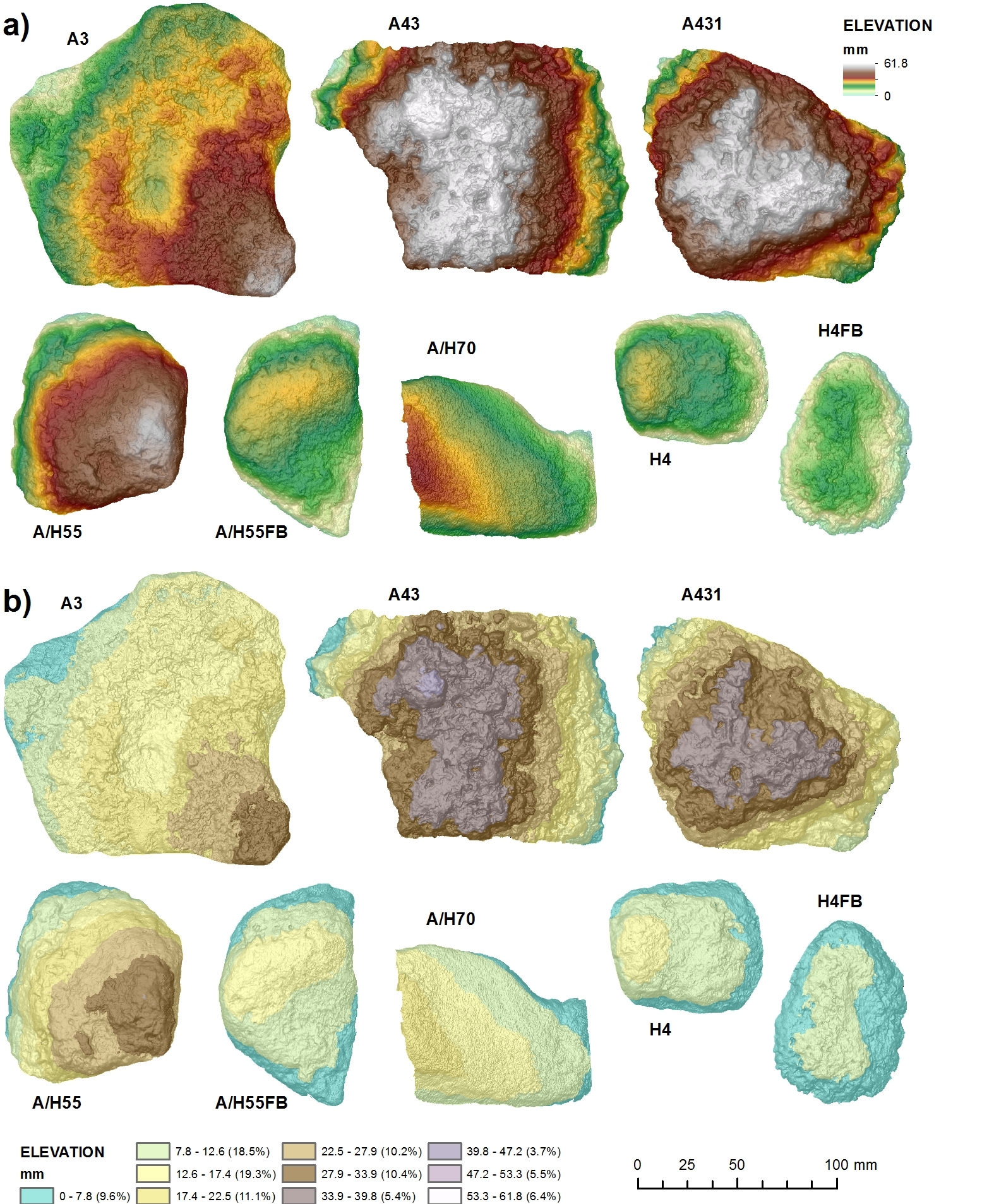

Supplement: S1 Fig — Digital Surface Models showing the elevation distribution in the stone tools faces. a) Continuous distribution of the elevation. b) Elevation distribution classified in ten groups defined by the statistical natural breaks of the data according to the Jenk´s method (ArcGIS 10.2.1). (JPG) [file pone.0121613.s001.jpg]

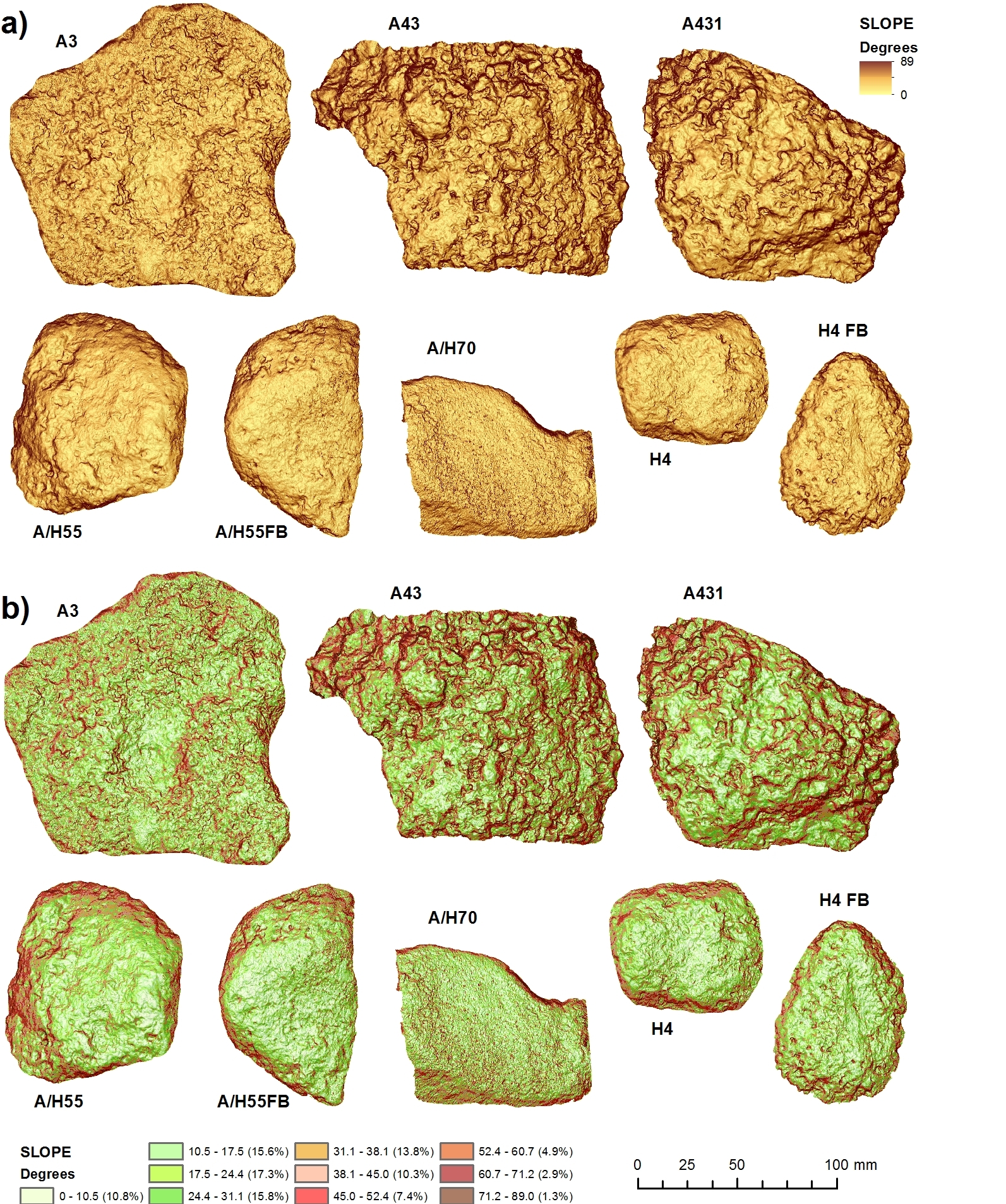

Supplement: S2 Fig — Digital Surface Models showing the slope distribution in the stone tools faces. Slope measures the rate of change of elevation in the direction of steepest descent. a) Continuous distribution of the slope. b) Slope distribution classified in ten groups defined by the statistical natural breaks of the data according to the Jenk´s method (ArcGIS 10.2.1). (JPG) [file pone.0121613.s002.jpg]

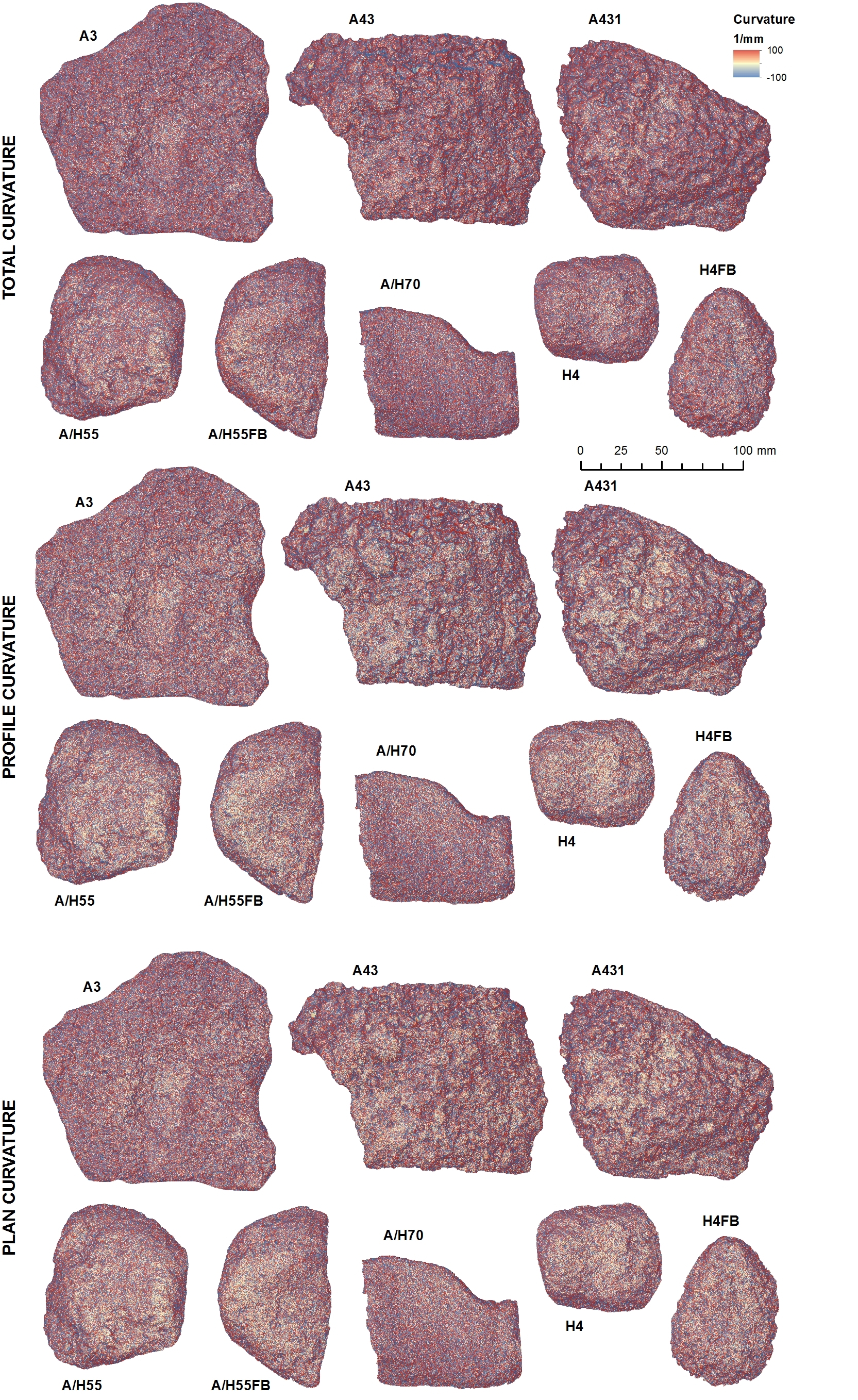

Supplement: S3 Fig — Digital Surface Models showing the curvature distribution in the stone tools faces. Curvature is the second derivate of the elevation, calculated following the steepest direction (profile curvature), its perpendicular (plan curvature), or a sum of both (tangential or total curvature). (JPG) [file pone.0121613.s003.jpg]

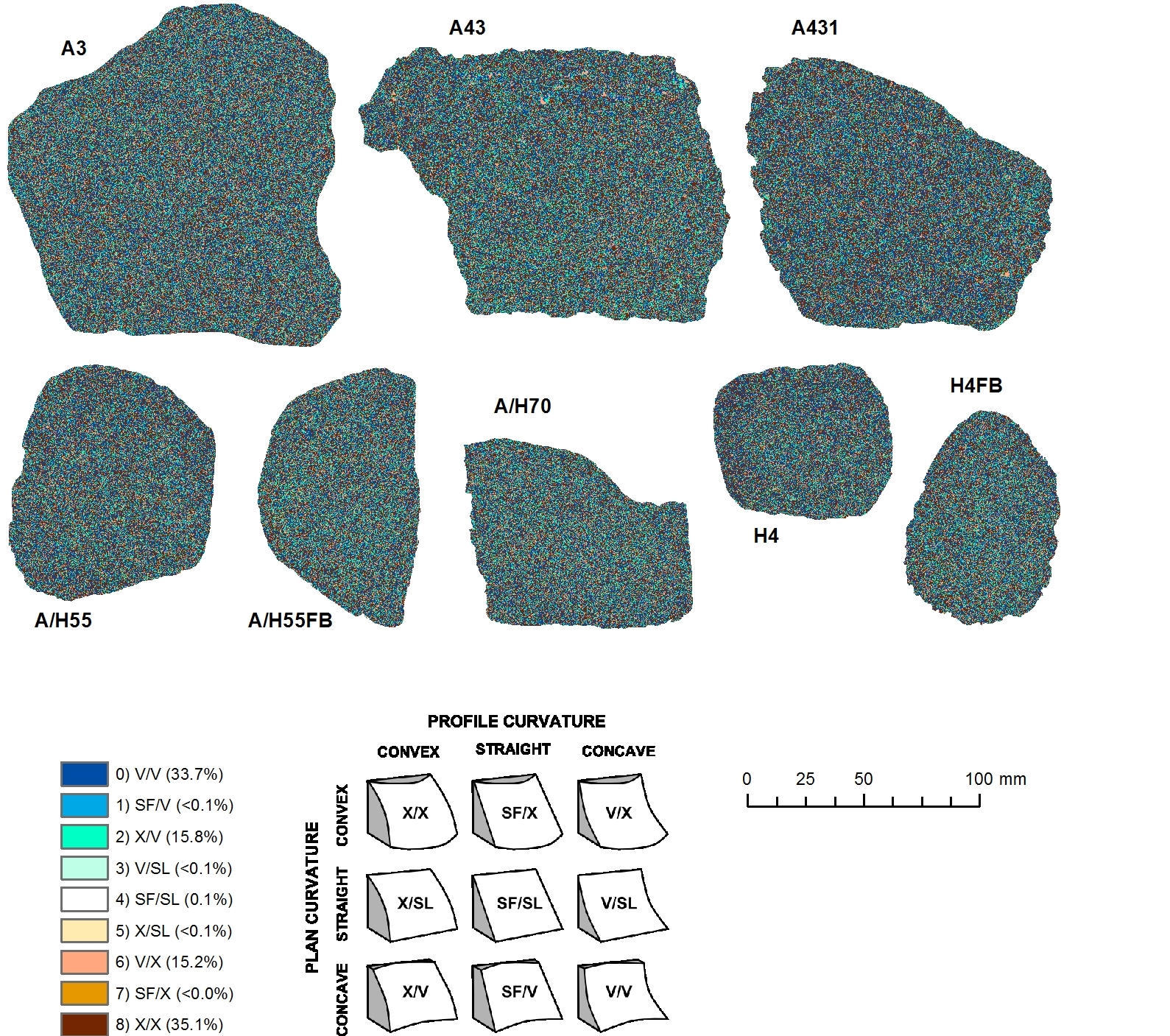

Supplement: S4 Fig — Curvature classification of the stone tools topography based on Dikau 1989, using profile and plan curvatures. (JPG) [file pone.0121613.s004.jpg]

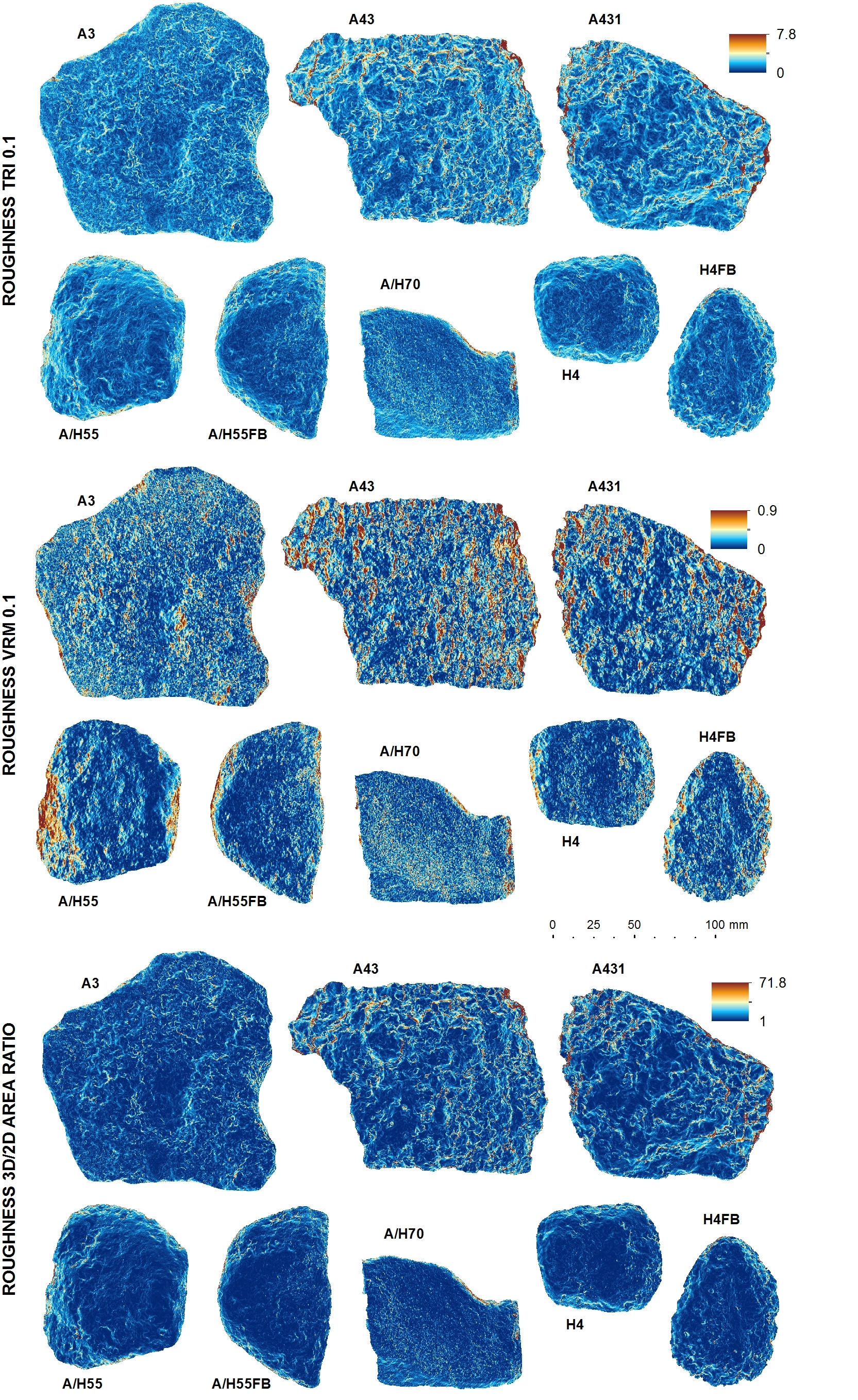

Supplement: S5 Fig — Digital Surface Models showing the roughness distribution in the stone tools faces, estimated through the Terrain Ruggedness Index (TRI), the Vector Ruggedness Measure (VRM), and the 3D/2D area ratio. Roughness models calculated considering the minimum neighborhood (radius = 0.1 mm). (JPG) [file pone.0121613.s005.jpg]

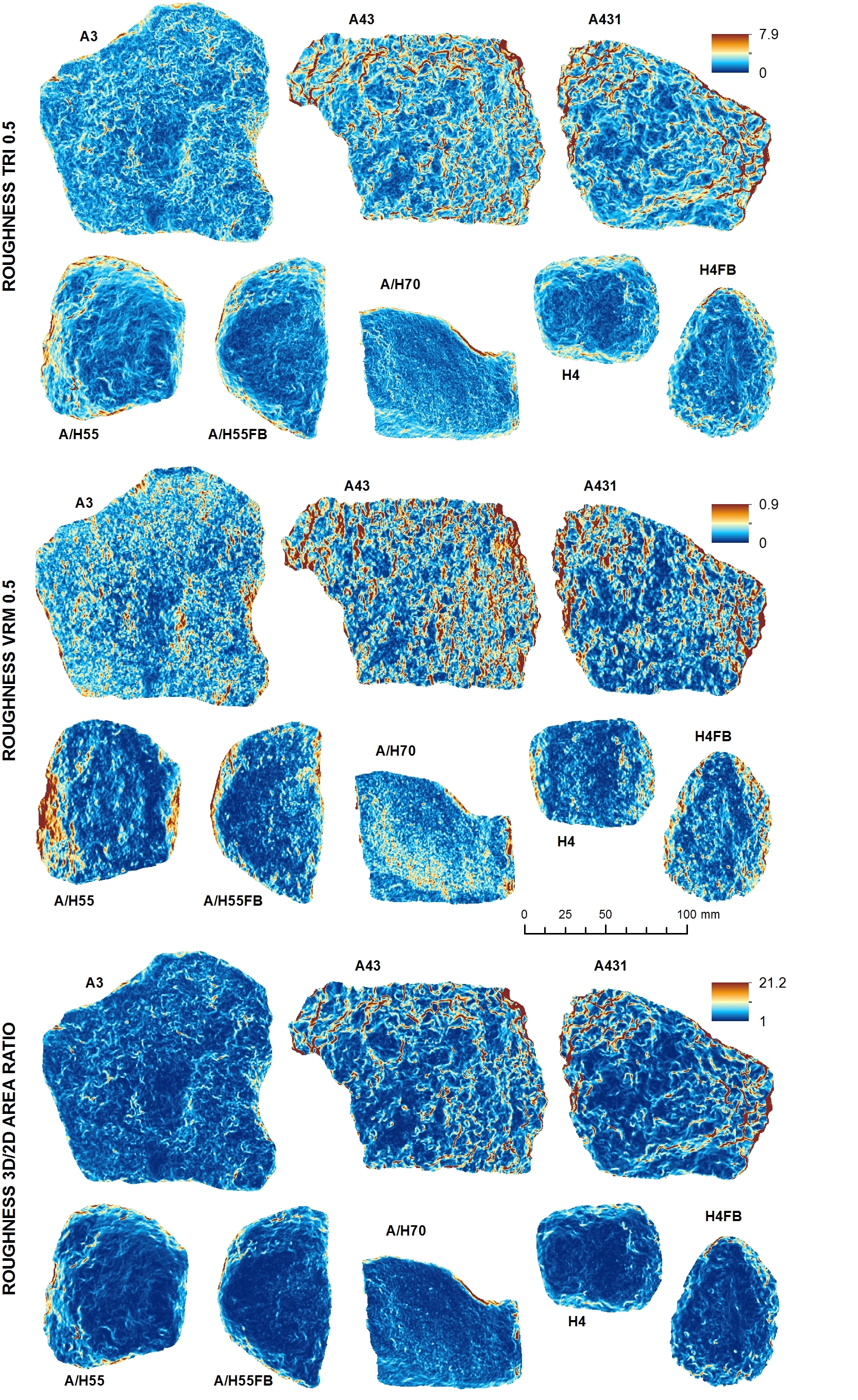

Supplement: S6 Fig — Digital Surface Models showing the roughness distribution in the stone tools faces, estimated through the Terrain Ruggedness Index (TRI), the Vector Ruggedness Measure (VRM), and the 3D/2D area ratio. Roughness models calculated considering a wider local neighborhood (radius = 0.5 mm). (JPG) [file pone.0121613.s006.jpg]

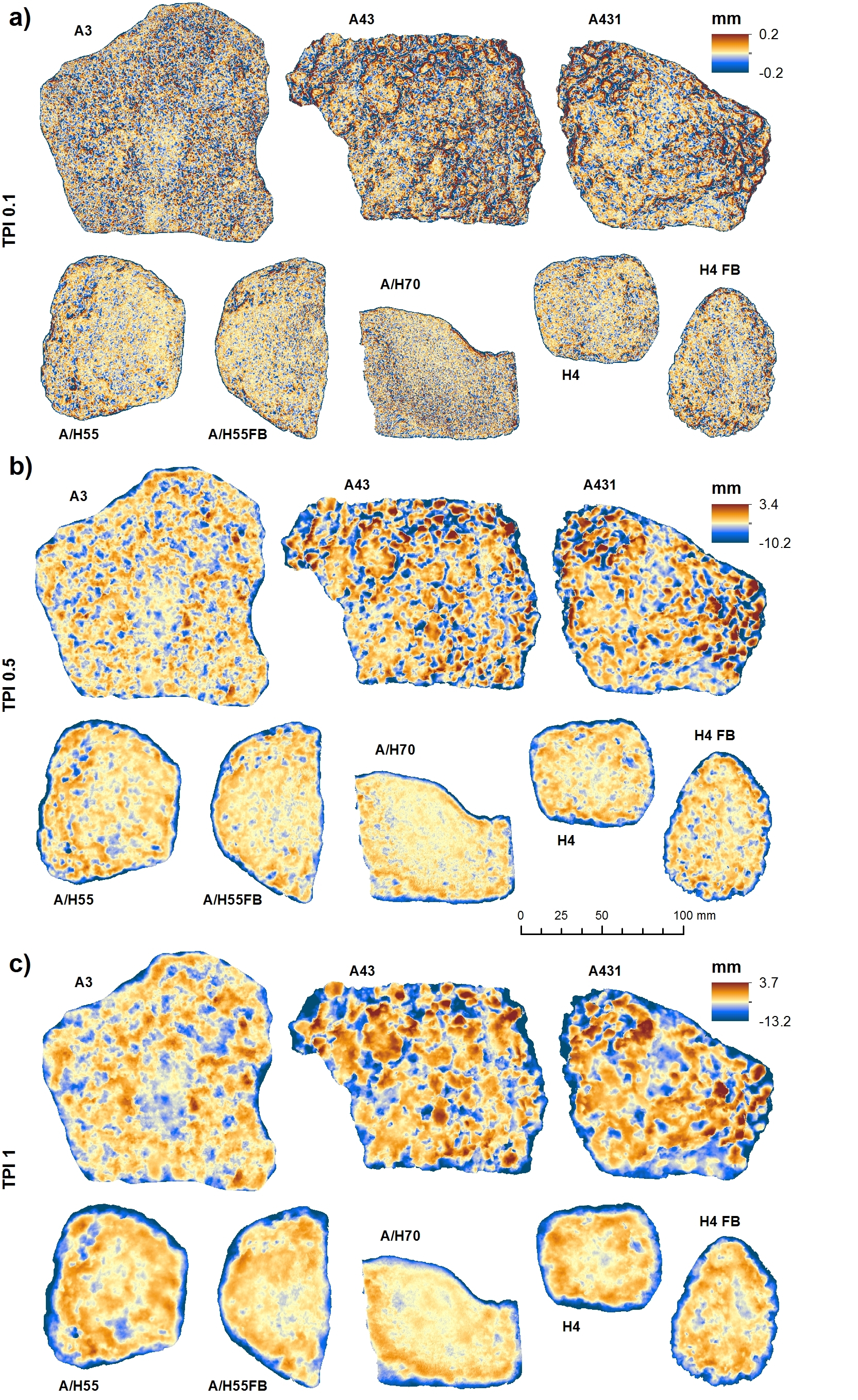

Supplement: S7 Fig — Digital Surface Models showing the value distribution of the Topographic Position Index in the stone tools faces, estimated considering neighborhood radius of 0.1 mm (a), 0.5 mm (b) and 1 mm (c). (JPG) [file pone.0121613.s007.jpg]
