# Supplementary material for: First GIS Analysis of Modern Stone Tools Used by Wild Chimpanzees (Pan troglodytes verus) in Bossou, Guinea, West Africa
Source: PLoS One. 2015 Mar 20;10(3):e0121613. doi: 10.1371/journal.pone.0121613 (PMC4368754; doi:10.1371/journal.pone.0121613)
Supplement: S1 Table — Elevation (mm), slope (degrees) and roughness (dimensionless) basic statistics of the stone tools, estimated from DSM. (DOC) [file pone.0121613.s008.doc]

| ***VARIABLE*** | ***ANVIL*** | ***COUNT*** | ***AREA*** | ***MIN*** | ***MAX*** | ***RANGE*** | ***MEAN*** | ***STD*** | ***SUM*** |
| --- | --- | --- | --- | --- | --- | --- | --- | --- | --- |
|  |  |  |  |  |  |  |  |  |  |
| **ELEVATION** |  |  |  |  |  |  |  |  |  |
|  | A3 | 1660422 | 16604.2 | 0.00 | 32.82 | 32.82 | 16.97 | 5.61 | 28169510.41 |
|  | A43 | 1428161 | 14281.6 | 0.00 | 41.40 | 21.04 | 26.83 | 8.89 | 67397166.59 |
|  | A431 | 1164053 | 11640.5 | 0.00 | 38.63 | 38.63 | 26.84 | 7.03 | 31247957.91 |
|  | A/H55 | 721738 | 7217.4 | 0.00 | 33.03 | 33.03 | 21.73 | 7.26 | 15684688.07 |
|  | A/H55FB | 596128 | 5961.3 | 0.00 | 16.38 | 16.38 | 10.56 | 3.42 | 6296636.27 |
|  | A/H70 | 630182 | 6301.8 | 0.00 | 22.16 | 22.15 | 13.04 | 3.94 | 8220333.26 |
|  | H4 | 442594 | 4425.9 | 0.00 | 15.53 | 15.53 | 9.67 | 3.11 | 4278580.67 |
|  | H4FB | 465914 | 4659.1 | 0.00 | 10.84 | 10.84 | 6.79 | 2.51 | 3164816.28 |
|  |  |  |  |  |  |  |  |  |  |
| **SLOPE** |  |  |  |  |  |  |  |  |  |
|  | A3 | 1660422 | 16604.2 | 0.03 | 84.77 | 84.74 | 27.50 | 13.93 | 45664387.33 |
|  | A43 | 1428161 | 14281.6 | 0.04 | 89.02 | 88.98 | 34.49 | 17.53 | 49251221.97 |
|  | A431 | 1164053 | 11640.5 | 0.03 | 88.95 | 88.92 | 33.05 | 17.53 | 38474971.85 |
|  | A/H55 | 721738 | 7217.4 | 0.03 | 86.57 | 86.54 | 27.77 | 14.62 | 20043536.88 |
|  | A/H55FB | 596128 | 5961.3 | 0.02 | 81.20 | 81.18 | 23.94 | 14.28 | 14269482.13 |
|  | A/H70 | 630182 | 6301.8 | 0.04 | 83.77 | 83.73 | 26.13 | 14.24 | 16466093.41 |
|  | H4 | 442594 | 4425.9 | 0.00 | 78.92 | 78.92 | 25.25 | 14.11 | 11176313.62 |
|  | H4FB | 465914 | 4659.1 | 0.04 | 83.76 | 83.72 | 25.73 | 14.55 | 11987358.58 |
|  |  |  |  |  |  |  |  |  |  |
| **ROUGHNESS** |  |  |  |  |  |  |  |  |  |
|  | **VRM01** |  |  |  |  |  |  |  |  |
|  | A3 | 1660422 | 16604.22 | 0.000039 | 0.819 | 0.819 | 0.081 | 0.085 | 134123.183 |
|  | A43 | 1428161 | 14281.61 | 0.000051 | 0.971 | 0.971 | 0.118 | 0.135 | 168772.434 |
|  | A431 | 1164053 | 11640.53 | 0.000017 | 0.965 | 0.965 | 0.099 | 0.131 | 115616.346 |
|  | A/H55 | 721738 | 7217.38 | 0.000008 | 0.935 | 0.935 | 0.075 | 0.099 | 54378.314 |
|  | A/H55FB | 596128 | 5961.28 | 0.000008 | 0.778 | 0.778 | 0.060 | 0.081 | 35944.011 |
|  | A/H70 | 630182 | 6301.82 | 0.000018 | 0.859 | 0.859 | 0.074 | 0.082 | 46842.211 |
|  | H4 | 442594 | 4425.94 | 0.000005 | 0.723 | 0.723 | 0.053 | 0.069 | 23620.138 |
|  | H4FB | 465914 | 4659.14 | 0.000017 | 0.817 | 0.817 | 0.077 | 0.091 | 35760.559 |
|  |  |  |  |  |  |  |  |  |  |
|  | **TRI01** |  |  |  |  |  |  |  |  |
|  | A3 | 1660422 | 16604.22 | 0.000503 | 1.443 | 1.442 | 0.045 | 0.031 | 75411.459 |
|  | A43 | 1428161 | 14281.61 | 0.000218 | 7.807 | 7.806 | 0.070 | 0.114 | 100609.083 |
|  | A431 | 1164053 | 11640.53 | 0.000195 | 6.563 | 6.563 | 0.073 | 0.148 | 85266.875 |
|  | A/H55 | 721738 | 7217.38 | 0.000293 | 2.182 | 2.182 | 0.046 | 0.042 | 33152.484 |
|  | A/H55FB | 596128 | 5961.28 | 0.000185 | 0.540 | 0.540 | 0.039 | 0.033 | 23316.639 |
|  | A/H70 | 630182 | 6301.82 | 0.000507 | 1.849 | 1.848 | 0.045 | 0.040 | 28555.077 |
|  | H4 | 442594 | 4425.94 | 0.000539 | 0.428 | 0.428 | 0.040 | 0.030 | 17568.840 |
|  | H4FB | 465914 | 4659.14 | 0.000238 | 1.300 | 1.300 | 0.042 | 0.038 | 19659.246 |
|  |  |  |  |  |  |  |  |  |  |
|  | **3D2D AREA RATIO** | |  |  |  |  |  |  |  |
|  | A3 | 1660422 | 16604.22 | 1.000116 | 8.923 | 7.923 | 1.204 | 0.244 | 1998531.926 |
|  | A43 | 1428161 | 14281.61 | 1.000074 | 49.620 | 48.620 | 1.450 | 0.963 | 2071207.412 |
|  | A431 | 1164053 | 11640.53 | 1.000075 | 44.230 | 43.230 | 1.463 | 1.196 | 1703335.188 |
|  | A/H55 | 721738 | 7217.38 | 1.000035 | 20.557 | 19.557 | 1.222 | 0.355 | 881711.667 |
|  | A/H55FB | 596128 | 5961.28 | 1.000030 | 5.776 | 4.776 | 1.170 | 0.258 | 697340.774 |
|  | A/H70 | 630182 | 6301.82 | 1.000178 | 10.432 | 9.432 | 1.200 | 0.283 | 756119.371 |
|  | H4 | 442594 | 4425.94 | 1.000108 | 4.664 | 3.664 | 1.176 | 0.228 | 520320.316 |
|  | H4FB | 465914 | 4659.14 | 1.000090 | 8.104 | 7.104 | 1.194 | 0.303 | 556506.940 |

**Table S1. DSM basic statistics of stone tools**. Elevation (mm), slope (degrees) and roughness (dimensionless) basic statistics of the stone tools, estimated from DSM.
