# Supplementary material for: First GIS Analysis of Modern Stone Tools Used by Wild Chimpanzees (Pan troglodytes verus) in Bossou, Guinea, West Africa
Source: PLoS One. 2015 Mar 20;10(3):e0121613. doi: 10.1371/journal.pone.0121613 (PMC4368754; doi:10.1371/journal.pone.0121613)
Supplement: S2 Table — Percentage of curvature classes in the depressions and ridges for every stone tool face. (DOC) [file pone.0121613.s009.doc]

|  |  |  |  | ***Depression*** | ***Ridge*** | |  | |  |  |  | ***Depression*** | ***Ridge*** |
| --- | --- | --- | --- | --- | --- | --- | --- | --- | --- | --- | --- | --- | --- |
|  | ***CURVATURE CLASS*** | | ***%*** | ***%*** | ***%*** |  | |  | ***CURVATURE CLASS*** | | ***%*** | ***%*** | ***%*** |
| ***A3*** | 0 | V/V | 33.77 | 36.81 | 29.64 |  | | ***A/H55FB*** | 0 | V/V | 32.67 | 36.30 | 28.64 |
|  | 1 | SF/V | 0.02 | 0.02 | 0.02 |  | |  | 1 | SF/V | 0.03 | 0.03 | 0.03 |
|  | 2 | X/V | 15.93 | 15.54 | 16.47 |  | |  | 2 | X/V | 17.01 | 16.36 | 17.73 |
|  | 3 | V/SL | 0.02 | 0.02 | 0.02 |  | |  | 3 | V/SL | 0.03 | 0.02 | 0.03 |
|  | 4 | SF/SL | 0.06 | 0.10 | 0.00 |  | |  | 4 | SF/SL | 0.09 | 0.18 | 0.00 |
|  | 5 | X/SL | 0.02 | 0.02 | 0.02 |  | |  | 5 | X/SL | 0.03 | 0.02 | 0.03 |
|  | 6 | V/X | 15.94 | 16.24 | 15.53 |  | |  | 6 | V/X | 16.11 | 16.04 | 16.19 |
|  | 7 | SF/X | 0.02 | 0.02 | 0.02 |  | |  | 7 | SF/X | 0.03 | 0.02 | 0.03 |
|  | 8 | X/X | 34.22 | 31.23 | 38.28 |  | |  | 8 | X/X | 34.01 | 31.02 | 37.31 |
|  |  |  |  |  |  |  | |  |  |  |  |  |  |
| ***A43*** | 0 | V/V | 32.69 | 36.94 | 27.54 |  | | ***A/H70*** | 0 | V/V | 33.27 | 38.85 | 26.55 |
|  | 1 | SF/V | 0.02 | 0.02 | 0.02 |  | |  | 1 | SF/V | 0.02 | 0.02 | 0.02 |
|  | 2 | X/V | 16.63 | 16.23 | 17.11 |  | |  | 2 | X/V | 16.63 | 16.20 | 17.14 |
|  | 3 | V/SL | 0.02 | 0.03 | 0.02 |  | |  | 3 | V/SL | 0.02 | 0.02 | 0.02 |
|  | 4 | SF/SL | 0.07 | 0.13 | 0.00 |  | |  | 4 | SF/SL | 0.09 | 0.16 | 0.01 |
|  | 5 | X/SL | 0.03 | 0.02 | 0.03 |  | |  | 5 | X/SL | 0.02 | 0.02 | 0.02 |
|  | 6 | V/X | 15.36 | 16.19 | 14.34 |  | |  | 6 | V/X | 16.11 | 15.89 | 16.39 |
|  | 7 | SF/X | 0.02 | 0.02 | 0.02 |  | |  | 7 | SF/X | 0.02 | 0.02 | 0.02 |
|  | 8 | X/X | 35.16 | 30.42 | 40.91 |  | |  | 8 | X/X | 33.83 | 28.82 | 39.85 |
|  |  |  |  |  |  |  | |  |  |  |  |  |  |
| ***A431*** | 0 | V/V | 34.93 | 38.10 | 30.81 |  | | ***H4*** | 0 | V/V | 35.11 | 38.49 | 31.22 |
|  | 1 | SF/V | 0.02 | 0.02 | 0.03 |  | |  | 1 | SF/V | 0.03 | 0.03 | 0.03 |
|  | 2 | X/V | 14.44 | 14.11 | 14.88 |  | |  | 2 | X/V | 14.59 | 14.40 | 14.80 |
|  | 3 | V/SL | 0.02 | 0.02 | 0.02 |  | |  | 3 | V/SL | 0.03 | 0.02 | 0.03 |
|  | 4 | SF/SL | 0.07 | 0.12 | 0.00 |  | |  | 4 | SF/SL | 0.10 | 0.18 | 0.00 |
|  | 5 | X/SL | 0.02 | 0.02 | 0.03 |  | |  | 5 | X/SL | 0.03 | 0.02 | 0.04 |
|  | 6 | V/X | 13.42 | 14.23 | 12.36 |  | |  | 6 | V/X | 13.87 | 14.31 | 13.36 |
|  | 7 | SF/X | 0.02 | 0.02 | 0.03 |  | |  | 7 | SF/X | 0.03 | 0.03 | 0.03 |
|  | 8 | X/X | 37.04 | 33.35 | 41.85 |  | |  | 8 | X/X | 36.23 | 32.52 | 40.49 |
|  |  |  |  |  |  |  | |  |  |  |  |  |  |
| ***A/H55*** | 0 | V/V | 34.42 | 37.07 | 31.52 |  | | ***H4FB*** | 0 | V/V | 33.12 | 36.67 | 29.09 |
|  | 1 | SF/V | 0.03 | 0.03 | 0.03 |  | |  | 1 | SF/V | 0.03 | 0.03 | 0.03 |
|  | 2 | X/V | 15.07 | 14.59 | 15.60 |  | |  | 2 | X/V | 16.48 | 15.79 | 17.27 |
|  | 3 | V/SL | 0.03 | 0.02 | 0.03 |  | |  | 3 | V/SL | 0.03 | 0.02 | 0.03 |
|  | 4 | SF/SL | 0.09 | 0.16 | 0.00 |  | |  | 4 | SF/SL | 0.11 | 0.21 | 0.00 |
|  | 5 | X/SL | 0.02 | 0.02 | 0.03 |  | |  | 5 | X/SL | 0.03 | 0.03 | 0.02 |
|  | 6 | V/X | 14.66 | 14.80 | 14.52 |  | |  | 6 | V/X | 15.72 | 15.75 | 15.69 |
|  | 7 | SF/X | 0.03 | 0.03 | 0.03 |  | |  | 7 | SF/X | 0.03 | 0.03 | 0.03 |
|  | 8 | X/X | 35.65 | 33.27 | 38.25 |  | |  | 8 | X/X | 34.46 | 31.47 | 37.85 |

**Table S2. Curvature characteristics of depressions and ridges**. Percentage of curvature classes in the depressions and ridges for every stone tool face.
