# Supplementary material for: First GIS Analysis of Modern Stone Tools Used by Wild Chimpanzees (Pan troglodytes verus) in Bossou, Guinea, West Africa
Source: PLoS One. 2015 Mar 20;10(3):e0121613. doi: 10.1371/journal.pone.0121613 (PMC4368754; doi:10.1371/journal.pone.0121613)
Supplement: S3 Table — (DOC) [file pone.0121613.s010.doc]

|  | **Value** | **Area** | **Min** | **Max** | **Range** | **Mean** | **STD** | **Sum** |
| --- | --- | --- | --- | --- | --- | --- | --- | --- |
| **Elevation** | 1, Depression | 39062.0 | 0.00 | 40.62 | 40.62 | 17.53 | 9.13 | 68465137.04 |
|  | 2, Ridge | 31938.9 | 3.12 | 41.40 | 38.28 | 20.93 | 9.38 | 66839539.15 |
| **Slope** | 1, Depression | 39165.5 | 0.00 | 88.97 | 88.97 | 31.60 | 16.63 | 123767730.42 |
|  | 2, Ridge | 31939.3 | 0.00 | 89.02 | 89.01 | 26.17 | 14.51 | 83592406.41 |
| **Roughness** |  |  |  |  |  |  |  |  |
| **VRM01** | 1, Depression | 39165.3 | 0.000000 | 0.97 | 0.97 | 0.10 | 0.12 | 387150.53 |
|  | 2, Ridge | 31939.3 | 0.000008 | 0.93 | 0.93 | 0.07 | 0.09 | 228095.18 |
| **TRI01** | 1, Depression | 39165.3 | 0.000000 | 6.56 | 6.56 | 0.06 | 0.10 | 243724.52 |
|  | 2, Ridge | 31939.3 | 0.000221 | 7.81 | 7.81 | 0.04 | 0.06 | 139904.84 |
| **2D/3DAREA** | 1, Depression | 39165.3 | 1.000064 | 47.70 | 46.70 | 1.36 | 0.84 | 5327526.46 |
|  | 2, Ridge | 31939.3 | 1.000030 | 49.62 | 48.62 | 1.21 | 0.45 | 3859655.39 |

**Table S3. DSM basic statistics in the depression and ridges of the stone tools**.
