# Supplementary material for: First GIS Analysis of Modern Stone Tools Used by Wild Chimpanzees (Pan troglodytes verus) in Bossou, Guinea, West Africa
Source: PLoS One. 2015 Mar 20;10(3):e0121613. doi: 10.1371/journal.pone.0121613 (PMC4368754; doi:10.1371/journal.pone.0121613)
Supplement: S4 Table — (DOC) [file pone.0121613.s011.doc]

|  | ***ANVIL*** | **A3** |  | **A43** |  | **A431** |  | **A/H55** |  | **A/H55FB** | | **A/H70** |  | **H4** |  | **H4FB** |  |
| --- | --- | --- | --- | --- | --- | --- | --- | --- | --- | --- | --- | --- | --- | --- | --- | --- | --- |
|  | ***ZONE*** | **D** | **R** | **D** | **R** | **D** | **R** | **D** | **R** | **D** | **R** | **D** | **R** | **D** | **R** | **D** | **R** |
| **Elevation** | *Min* | 0.00 | 4.74 | 0.0 | 9.1 | 0.24 | 9.77 | 0.08 | 7.51 | 0.00 | 4.85 | 0.00 | 4.34 | 0.05 | 5.34 | 0.00 | 3.12 |
|  | *Max* | 32.11 | 32.82 | 40.6 | 41.4 | 37.75 | 38.63 | 32.57 | 33.03 | 16.17 | 16.38 | 21.71 | 22.16 | 14.93 | 15.53 | 10.31 | 10.84 |
|  | *Range* | 32.11 | 28.08 | 40.6 | 32.4 | 37.51 | 28.86 | 32.49 | 25.53 | 16.17 | 11.53 | 21.71 | 17.81 | 14.88 | 10.19 | 10.31 | 7.72 |
|  | *Mean* | 16.00 | 18.27 | 24.6 | 29.6 | 24.59 | 29.82 | 19.78 | 23.89 | 9.19 | 12.11 | 12.09 | 14.20 | 8.39 | 11.15 | 5.62 | 8.15 |
|  | *STD* | 5.51 | 5.48 | 9.2 | 7.7 | 7.18 | 5.54 | 7.75 | 5.93 | 3.41 | 2.65 | 3.83 | 3.73 | 3.19 | 2.21 | 2.55 | 1.61 |
| **Slope** | *Min* | 0.03 | 0.08 | 0.0 | 0.0 | 0.06 | 0.03 | 0.03 | 0.03 | 0.02 | 0.02 | 0.07 | 0.04 | 0.02 | 0.00 | 0.04 | 0.05 |
|  | *Max* | 84.66 | 84.77 | 89.0 | 89.0 | 88.95 | 88.87 | 86.57 | 85.30 | 81.20 | 78.20 | 83.77 | 80.28 | 78.92 | 77.22 | 83.76 | 78.30 |
|  | *Range* | 84.63 | 84.69 | 88.9 | 89.0 | 88.89 | 88.84 | 86.54 | 85.27 | 81.18 | 78.18 | 83.70 | 80.24 | 78.90 | 77.22 | 83.72 | 78.25 |
|  | *Mean* | 28.43 | 26.25 | 37.3 | 31.1 | 35.20 | 30.26 | 31.08 | 24.16 | 27.79 | 19.67 | 28.03 | 23.84 | 28.87 | 21.10 | 29.60 | 21.33 |
|  | *STD* | 14.28 | 13.34 | 18.0 | 16.3 | 18.15 | 16.26 | 15.85 | 12.15 | 15.54 | 11.28 | 14.99 | 12.93 | 15.20 | 11.41 | 15.59 | 11.83 |
| **Roughness** |  |  |  |  |  |  |  |  |  |  |  |  |  |  |  |  |  |
| **VRM01** | *Min* | 0.00004 | 0.00004 | 0.00005 | 0.00006 | 0.00002 | 0.00002 | 0.00002 | 0.00001 | 0.00003 | 0.00001 | 0.00002 | 0.00013 | 0.00000 | 0.00003 | 0.00002 | 0.00005 |
|  | *Max* | 0.82 | 0.74 | 0.97 | 0.91 | 0.96 | 0.93 | 0.93 | 0.81 | 0.78 | 0.62 | 0.86 | 0.81 | 0.72 | 0.69 | 0.82 | 0.75 |
|  | *Range* | 0.82 | 0.74 | 0.97 | 0.91 | 0.96 | 0.93 | 0.93 | 0.81 | 0.78 | 0.62 | 0.86 | 0.81 | 0.72 | 0.69 | 0.82 | 0.75 |
|  | *Mean* | 0.09 | 0.07 | 0.13 | 0.10 | 0.11 | 0.08 | 0.09 | 0.06 | 0.08 | 0.04 | 0.08 | 0.07 | 0.06 | 0.04 | 0.10 | 0.05 |
|  | *STD* | 0.09 | 0.08 | 0.15 | 0.12 | 0.15 | 0.11 | 0.11 | 0.07 | 0.10 | 0.05 | 0.09 | 0.08 | 0.08 | 0.05 | 0.10 | 0.06 |
| **TRI01** | *Min* | 0.00050 | 0.00067 | 0.00022 | 0.00046 | 0.00020 | 0.00022 | 0.00033 | 0.00029 | 0.00019 | 0.00053 | 0.00073 | 0.00051 | 0.00068 | 0.00054 | 0.00049 | 0.00024 |
|  | *Max* | 1.37 | 1.44 | 6.00 | 7.81 | 6.56 | 6.47 | 2.18 | 1.57 | 0.54 | 0.48 | 1.85 | 1.17 | 0.43 | 0.39 | 1.30 | 0.37 |
|  | *Range* | 1.37 | 1.44 | 6.00 | 7.81 | 6.56 | 6.47 | 2.18 | 1.57 | 0.54 | 0.48 | 1.85 | 1.17 | 0.43 | 0.39 | 1.30 | 0.37 |
|  | *Mean* | 0.05 | 0.04 | 0.08 | 0.06 | 0.09 | 0.06 | 0.05 | 0.04 | 0.05 | 0.03 | 0.05 | 0.04 | 0.05 | 0.03 | 0.05 | 0.03 |
|  | *STD* | 0.03 | 0.03 | 0.14 | 0.07 | 0.18 | 0.09 | 0.05 | 0.03 | 0.04 | 0.02 | 0.05 | 0.03 | 0.03 | 0.02 | 0.04 | 0.02 |
| **2D/3D** | *Min* | 1.00013 | 1.00012 | 1.00012 | 1.00007 | 1.00014 | 1.00007 | 1.00006 | 1.00004 | 1.00007 | 1.00003 | 1.00018 | 1.00028 | 1.00011 | 1.00023 | 1.00009 | 1.00013 |
| **Area Ratio** | *Max* | 8.72 | 8.92 | 47.7 | 49.6 | 44.23 | 41.08 | 20.56 | 10.08 | 5.78 | 4.21 | 10.43 | 6.78 | 4.66 | 3.83 | 8.10 | 4.43 |
|  | *Range* | 7.71 | 7.92 | 46.7 | 48.6 | 43.23 | 40.08 | 19.56 | 9.08 | 4.78 | 3.21 | 9.43 | 5.78 | 3.66 | 2.83 | 7.10 | 3.43 |
|  | *Mean* | 1.22 | 1.18 | 1.6 | 1.3 | 1.57 | 1.32 | 1.29 | 1.14 | 1.23 | 1.10 | 1.24 | 1.15 | 1.23 | 1.11 | 1.26 | 1.12 |
|  | *STD* | 0.26 | 0.22 | 1.2 | 0.6 | 1.43 | 0.78 | 0.45 | 0.18 | 0.32 | 0.14 | 0.35 | 0.17 | 0.27 | 0.14 | 0.37 | 0.16 |
|  |  |  |  |  |  |  |  |  |  |  |  |  |  |  |  |  |  |
|  |  |  | D, | Depressions | |  |  |  |  |  |  |  |  |  |  |  |  |
|  |  |  | R, | Ridges |  |  |  |  |  |  |  |  |  |  |  |  |  |

**Table S4. DSM basic statistics in the depression and ridges for every stone tool**
