# Supplementary material for: First GIS Analysis of Modern Stone Tools Used by Wild Chimpanzees (Pan troglodytes verus) in Bossou, Guinea, West Africa
Source: PLoS One. 2015 Mar 20;10(3):e0121613. doi: 10.1371/journal.pone.0121613 (PMC4368754; doi:10.1371/journal.pone.0121613)
Supplement: S1 Text — Morphometric analysis and spatial pattern analysis from visual identification. (DOCX) [file pone.0121613.s012.docx]

**GIS Methodology**

*Morphometric analysis*

Three-dimensional digital models of percussive tool surfaces were created using the triangulation-based 3D laser scanner Next Engine. The pieces were scanned using 3 shoots at macro mode (0.127 mm resolution per shoot). Resulting point clouds were exported to a raster GIS format, where data were rasterized at 0.1 mm resolution. Missing data in the initial models were filled using a spline interpolation method.

Resulting Digital Elevation Models (DEM) of each tool face (N=8, see Figure 7) were used for surface morphometric analysis of topographic attributes calculated with GIS (ArcGIS 10.1 and SAGA 2.1). Several Digital Surface Models (DSM) were calculated from elevation data, such as primary and secondary derivates (i.e. slope, aspect and curvatures) or hillshading models [48]-[49]. These DSM were used for a first basic morphometric analysis of the stone tools surfaces. Subsequently, roughness, topographic position and relative depth DSM were specifically applied for the automatic identification and interpretation of the use-wear features.

Roughness measures the variability or heterogeneity of a topographic surface, and is a key property to identify areas of use (e.g. polish) at the battered surface. Several techniques have been proposed to calculate roughness [50]-[56], but there is no standard method as yet. In this work, we have tested three widely used methods: Terrain Ruggedness Index (TRI), Vector Ruggedness Measure (VRM), and the 3D/2D area ratio. Terrain Roughness Index (TRI) calculates the sum change in elevation between a grid cell and its neighborhood, according to the algorithm by [40]. TRI is always ≥ 0 m, where 0 represents the minimum roughness. VRM measures roughness as the dispersion of vectors orthogonal to the surface within a specific neighborhood [50], [55]-[56]. This method captures variability in slope and aspect into a single measure, which is usually expressed from 0 (no terrain variation or lowest roughness) to 1 (complete terrain variation or maximum roughness). Finally, the 3D/2D area ratio is based on the differences when calculating the area in two or three dimensions. A sloped pixel covers a larger area than a flat one, and the difference is interpreted as the roughness. This index is described largely independent from scale [54], although it is highly dependent on the slope. In this work, this index has been calculated as the 3D/2D areas ratio, in such a way that the index ≥ 1, and 1 represents the minimum roughness.

In order to better characterize the areas of the tools with lowest roughness (which may correspond to polish use wear), roughness models were also calculated considering a wider local neighborhood (0.5 mm radius). TRI, VRM and 3D/2D area ratio roughness models were combined into a final model, which consisted in the normalization of the three models, and their addition into a unique model which was then normalized again. This final roughness model integrates the singularities of the three models into a single map where roughness varies from 0 (minimum roughness) to 1 (maximum roughness). From this map, polished areas showing the lowest roughness values were extracted on the basis of an arbitrary low value of <0.01.

An elevation residual analysis was also used in order to identify and map depressions and ridges of the battered surfaces. This analysis, so-called Topographic Position Index (TPI), calculates the difference between the elevation of a cell and the mean elevation in a neighborhood surrounding that cell. Neighborhood mean elevation is calculated using a moving window centered on the cell of interest. TPI positive values indicate that the cell is higher than its neighborhood, while negative values indicate the cell is lower. Topographic Position Index (TPI) was calculated as proposed by [57], which coincides with difference to the mean calculation (residual analysis) proposed by [48]. The bandwidth parameter for distance weighting is given as percentage of the (outer) radius. TPI is scale-dependent index, sensitive to local differences from regional elevations, allowing separation of upper slopes or ridges (where TPI is positive, i.e. the elevation of the cell is higher than the mean elevation of the neighborhood) from lower slopes or depressions (where TPI yields negative values, as the elevation of the cell is lower than the mean elevation of the neighborhood).

TPI enables mapping the spatial distribution of ridges and depressions, but does not provide data on the relative depth of depressions. To calculate this variable, firstly a Top Potential Surface or TPS model was interpolated for each battered surface. TPS represents the surface joining the highest points of the ridges. It was interpolated using a spline method, which bends a minimum curvature surface passing through all the highest ridges. Once this surface was interpolated, battered surface Digital Elevation Models (DEM) were subtracted from TPS models, hence obtaining the relative depth models for depressions in each artifact.

*Visual mapping and spatial pattern analysis*

Following protocols outlined by de la Torre et al [25], digital images were geo-referenced in a local Cartesian system using ArcGIS 10.1. Macroscopically-identified percussive marks were outlined over the images, and indexes such as area, perimeter, and distribution and size of the areas covered by percussion marks, were calculated to produce a spatial pattern of the use wear distribution along the tools.

Use wear was assessed calculating the percentage of the artefact covered by use-wear (PA index), largest use-wear (LUW), use wear density (D, obtained dividing the number of marks identified by the total plane area), and edge density (ED, sum of all use-wear perimeters divided by the total plane area). The mean shape of all percussive marks is represented by the MNSH index, compared with a standard circular shape (MNHS=1). The distribution of the use-wear along the surfaces was assessed calculating the distance of the marks to the geometric center of the artifact (DAC), and the distance to the edge (DAE). The directional trends and relative position of the marks was estimated using the standard deviational ellipses, calculating also standard distances along the X and Y axes (XstdD and YstdD). The EMNC (ellipse mean center) was used to estimate the relative position of the marks regarding the battered surface center (EMNC-AC) and to the tool edge (EMNC-AE), as well the distance between the ellipse mean center and the median center (EMNC-MDC) [37].
